# Evaluating the participatory processes within the OECD PaRIS project in Saskatchewan: lessons learned about recruitment and engagement

**DOI:** 10.1186/s12875-026-03288-4

**Published:** 2026-04-01

**Authors:** Udoka Okpalauwaekwe, Rachel Vanneste, Brenda Andreas, Mark Lees, Dawn Martin, Lisa Clatney, Paul Lendzyk, Aubrey Tollefson, Jennifer Kuzmicz, Kristin Foy, Nazeem Muhajarine, Vivian R. Ramsden, the PaRIS Saskatchewan Research Team, Reem Abdul Hadi, Bonnie Ast, Mahshid Bagheri, Shannon Bandura, Angela Baerwald, Michael Bayda, Candina Beaurivage, Doug Bell, Colleen Brockbank, Kristin Canart, Claire Chao Danielle Chartier, Darren Chew, Megan Clark, Jeannie Coe, Cathy Cole, Holly Anne Cook-Laliberte, Ettiene Crouse, Starr Davis, Christine Dawson, Armaan Dogra, John Dosman, Nicole Dubois, Kris Dutchak, Cara Fallis, Jill Farrukh, Eleanor Francis, Danielle Frost, Joanna Fynn, Hillary Gable, Asma Gargoum, Kali Gartner, Rachel Gough, Kevin Govender, Seema Goyal, Brendan Groat, Twila Grona, Sean Groves, Tracey Guselle, Joan Hamilton, Jacelyn Hanson, Jessica Harris, Boris Hencic, Martin Heroux, Alyx Holden, Caylee Holden, Carla Holinaty, Jason Hosain, Somto Ibezi, Hazel Javier, Rachel Johnson, Sylvie Jones, Rejina Kamrul, Ross Kerhoff, Matt Kushneriuk, Leung Kwok, Angela Lapetsky, Dag Lawale, Kathy Lawrence, Kevin Ledding, Crystal Litwin, Faith Lubansa, Cathy MacLean, Della Magnusson, Loreanne Manalac, Morris Markentin, Razawa Maroof, Radhika Marwah, Darcie McGonigle, Reid McGonigle, Meredith McKague, Robin McMaster, Andries Muller, Michael Nicholls, Solveig Nilson, Buchi Nwodo, Cindy Nylund, Meric Osman, Paula Paley, Cassandra Pancyr, Leane Pask, Jodi Parent, Payton Pederson, Rae Petrucha, Aaron Prystupa, Olivia Reis, Volker Rininsland, Jessica Rivas, Angela Robinson, Clara Rocha Michaels, Erika Roets, Wessel Roets, Bethany Rolfe, Ginger Ruddy, Isa Saidu, Easan Sasithary, Marty Salter, Herman Schalk van der Merwe, Crystal Semple, Erin Selzer, Tharshan Selestin, Nicole Shedden, Lesley Shoemaker, Mark Tarry, Beavan Talukdar, Arvinder Theathi, Gillian Treen, Aaron Vanderlot, Andrea Vasquez Camargo, Felecia Watson, Yan Wu, Cheryl Zagozeski, Sinisa Zerajic

**Affiliations:** 1 Department of Family Medicine, College of Medicine, University of Saskatchewan, Saskatoon, SK S7M 3Y5 Canada; 2 Regina Community Clinic, Regina, SK S4R 1J6 Canada; 3 Saskatoon Community Clinic, Saskatoon, SK S7K 2S2 Canada; 4 Wynyard Community Health Centre, Wynyard, SK S0A 4T0 Canada; 5 Mossbank and Lafleche Primary Healthcare Clinics, Lafleche, SK Canada; 6 Department of Family Medicine, College of Medicine, University of Saskatchewan, Saskatoon, SK S4P 2S5 Canada; 7 Department of Family Medicine, College of Medicine, University of Saskatchewan, Saskatoon, SK S0G 0G7 Canada; 8 College of Medicine, Community Health and Epidemiology, University of Saskatchewan, Saskatoon, SK S7M 3Y5 Canada

**Keywords:** OECD, Patient-Reported Indicator Surveys (PaRIS), Participatory Evaluation, Relational Equity, Primary Care, Saskatchewan

## Abstract

**Objectives:**

To evaluate the participatory recruitment and engagement processes used in the implementation of the Organisation for Economic Co-operation and Development (OECD) Patient-Reported Indicator Surveys (PaRIS) Project in Saskatchewan, Canada, and to identify lessons to inform future primary care data initiatives.

**Design:**

Participatory evaluation using a qualitative, interpretive descriptive approach, guided by a participatory evaluation framework grounded in relational equity.

**Setting:**

Ten purposively selected primary care clinics across Saskatchewan, including academic family medicine units, community health centres, a nurse practitioner–led clinic, and a northern group physician practice, representing urban, rural, remote, and Indigenous-serving areas in the province.

**Participants:**

Primary care practitioners (family physicians and nurse practitioners), clinic administrators and medical office assistants, and people with lived experience who were involved in PaRIS survey implementation and interpretation of clinic-level dashboards.

**Intervention:**

Clinic-level engagement with OECD PaRIS dashboards presenting aggregated patient-reported experience measures (PREMs) and patient-reported outcome measures (PROMs), supported through relational, trust-based engagement, feedback sessions, and participatory interpretation.

**Primary and secondary outcome measures:**

Perceived effectiveness of recruitment and engagement strategies; experiences of dashboard interpretation and use; identified facilitators, challenges, and opportunities for improvement in participatory primary care data initiatives.

**Results:**

Early, trust-based engagement fostered strong clinic participation, a sense of co-ownership of data, and increased willingness to use findings for reflection and quality improvement. Participants described the dashboards as validating and empowering, particularly when supported by contextualized narratives. Key facilitators included relational transparency, clinic-specific feedback, and iterative feedback loops. Challenges included visual complexity of dashboards, limited access to comparative data due to embargoes, time constraints for team-based reflection, and reduced accessibility for some digitally underserved patient groups. Participants identified opportunities to simplify data visualizations, tailor dissemination formats, and extend engagement timelines, particularly when working with Indigenous communities.

**Conclusions:**

Relationship-driven, participatory approaches can meaningfully enhance engagement with PREMs and PROMs in primary care. Embedding relational equity throughout recruitment, data interpretation, and feedback processes supports co-ownership of data and strengthens its relevance for local quality improvement. Our findings offer practical guidance for designing inclusive, culturally responsive primary care data initiatives.

**Supplementary Information:**

The online version contains supplementary material available at 10.1186/s12875-026-03288-4.

## Introduction

### Problem description

Primary care is the cornerstone of any effective health system, providing first contact, continuous, and comprehensive care [1, 2]. However, across Canada and particularly in Saskatchewan, this foundation has been under strain [2–4]. With a population of approximately 1.2 million, Saskatchewan faces primary care challenges including, shortages of primary care practitioners (PCPs), especially in rural and remote communities, primary care accessibility, coordination and continuity challenges [2, 5]. Over 13% of Canadians, including many in Saskatchewan, lack access to a regular PCP, with chronic diseases and frailty disproportionately affecting seniors, First Nations and Métis populations [2, 3, 6]. These issues have widened gaps in access, coordination, responsiveness, and equitable care delivery within the province's primary care landscape [7–9]. As a result, limited access to primary care has been associated with increased Emergency Department utilization, higher rates of avoidable hospitalizations, poorer chronic disease outcomes, and increased mortality, particularly among rural and underserved populations [6].

In response to these challenges, there has been a growing interest in approaches that incorporate patient-reported experience measures (PREMs) and patient-reported outcome measures (PROMs) to better understand care delivery from the patient’s perspective and inform local continuous quality improvement efforts within primary care settings.

### Available knowledge

A global push for health system reform has placed increasing emphasis on PREMs and PROMs to capture the perspectives of all those who use healthcare services, including PCP and patients [10–13]. In response to this, the Organisation for Economic Co-operation and Development (OECD) launched the Patient-Reported Indicator Surveys (PaRIS) initiative, which is an international effort aimed at standardizing the collection and use of patient-centered data in primary care settings [11, 12, 14]. The PaRIS Project extended beyond traditional patient satisfaction metrics by capturing specific aspects of how care is experienced in practice. These included whether or not providers spent sufficient time with patients, involved them in decision-making, communicated clearly, and treated them as a whole person rather than focusing solely on the disease. It also assesses patients’ experiences of access (e.g., wait times), perceived safety issues, and coordination of care across providers [15]. In addition, PaRIS captures patient-reported outcomes such as self-rated health, pain interference with daily activities, mental health (e.g., anxiety or depression), confidence in managing one’s health, and overall quality of life. Together, these measures provided a more comprehensive understanding of how primary care systems function from the patient’s perspective, particularly in relation to person-centered, relational, and coordinated care [15]. The OECD PaRIS’s instrument for patients used in this study was co-developed for this international initiative by the OECD and participating countries, including Canada. Although the full questionnaire has not been published in a peer-reviewed journal, the complete English-language questionnaire is publicly available on the OECD’s website (https://www.oecd.org/content/dam/oecd/en/about/programmes/patient-reported-indicator-surveys/PaRIS%20patient%20questionnaire.pdf). See Supplementary File A for more information on the PaRIS questionnaire).

Overall, PaRIS brought a new level of international collaboration by facilitating health systems in OECD participating countries to compare health indicators across countries to inform opportunities for improvement based on what mattered most to patients [13, 14]. In Canada, the OECD PaRIS initiative was the largest clinically-based primary care patient survey in the past decade, designed to amplify patient voices so as to better understand the current state of primary care, particularly from the perspective of patients’ experiences, care coordination, and health outcomes [14]. Even though Saskatchewan’s health system faces many challenges [2, 3, 6], our work on the OECD PaRIS Project stood out as being successful. Working collaboratively with a wide range of partners, including family physicians, nurse practitioners, people with lived experience (PWLE), health administrators, medical office assistants (MOA), decision makers, knowledge users, and academic researchers, we were able to launch the survey, reaching and exceeding our provincial targets for the project. Our approach, which focused on relationship-driven, participatory and inclusive engagement, saw 1,324 surveys from both patients (response rate at 39%) and PCPs (response rate at 54%) returned within a period of four months (July 1, 2024 to October 31, 2024).

### Study rationale

As healthcare systems increasingly look to PREMs and PROMs to guide planning and evaluation of services, questions about how to authentically engage both health care practitioners and people with lived experience in the process of data collection remain [1, 16, 17]. Traditional top-down recruitment processes often demonstrate low response rates and limited engagement, particularly in historically underserved or marginalized communities [17, 18]. In this context, Saskatchewan’s participatory approach offers some valuable insights [18, 19]. Within our approach, we nurtured “relational equity [20],” ensuring that every team member’s voice was heard, every voice mattered, and every voice was supported to be heard on all aspects of the research journey. As such, we were able to generate not only high participation rates but also a context-sensitive model of enhanced community-engaged research. As such, we were inspired and opted to evaluate our processes of participatory engagement to understand what worked, what didn’t and what is essential to informing future primary care research, especially within contexts facing similar systemic constraints.

### Specific objectives

To evaluate the participatory processes employed in the implementation of the OECD PaRIS Project in Saskatchewan. Specifically, we aspired to:

- Evaluate the recruitment and engagement strategies used to involve PCPs and people with lived experience (PWLE) across diverse clinical settings. This included examining how partners interpreted, applied, and responded to the OECD PaRIS dashboards, with a focus on learning, capacity-building, and local quality improvement.
- Identify the key facilitators and challenges experienced during the PaRIS rollout.
- Draw on lessons from this evaluation to inform future participatory initiatives within primary care settings.

## Methods

### Study design and framework

Our study employed a participatory evaluation framework informed by Fetterman’s [21] principles of evaluation. This approach emphasized shared ownership, inclusivity, and relational equity, ensuring that all members of the research team were actively involved in shaping the evaluation process [21]. The role of the evaluator in this process was that of a facilitator, working with the members of the research team to shape the processes of the evaluation [21].

### Processes and procedures

Our evaluation focused on how participants interacted with the OECD-PaRIS Project from design to data collection and interpretation of the OECD PaRIS dashboards, which summarized Patient-Reported Experience Measures (PREMs) and Patient-Reported Outcome Measures (PROMs) data at both local (primary care clinics) and provincial levels. Using this participatory lens [22, 23], we explored clinic-level engagement, perceived data relevance, data interpretation challenges, and opportunities for improvement.

### Context

Our study took place in Saskatchewan, one of ten Canadian provinces [2, 24]. Saskatchewan is uniquely marked by its vast geographic and demographic diversity. Primary care delivery in this province spans urban centers, rural and remote towns, and numerous Indigenous communities [2, 24, 25]. The first cycle of the PaRIS Project in Saskatchewan engaged ten primary care clinics as research partners, selected purposively to reflect this diversity in geography, organizational structure, and care delivery approach. Participating clinics included four academic teaching units affiliated with the University of Saskatchewan’s Department of Family Medicine (DFM), four interdisciplinary community health centres (CHCs) operating within cooperative or not-for-profit models, one independently operated nurse practitioner-led clinic, and one group physician practice. Together, these clinics serve heterogeneous patient populations, including urban inner-city residents, rural and remote communities, and Indigenous populations. Our partnering clinics varied in size, team composition, and scope of services, ranging from large interdisciplinary teams to smaller, clinician-led practices. Table 1 provides a descriptive summary of clinic characteristics, including practice model, geographic setting, team structure, and key patient population attributes.

**Table 1:** Participating clinic characteristics

Clinic Type	# of Clinics	Description
Department of Family Medicine (DFM)-Affiliated Clinics	4	Affiliated with the University of Saskatchewan, these clinics are located in urban, semi-urban, and rural areas. They offer full-scope primary care and emergency coverage, support undergraduate and postgraduate training, and serve diverse populations, including older adults, newcomers, and Indigenous patients. Teams include family physicians, NPs, RNs, residents, nurses, social workers, and admin staff. Catchment populations range from 6,000 to 20,000
Community Health Centres (CHCs)	4	These community-governed clinics (urban and rural) operate under cooperative or non-profit models. They focus on holistic care, equity, and the social determinants of health. Many serve populations with complex needs, such as individuals with mental health challenges, inner-city residents, newcomers, and those facing housing insecurity. Teams are interdisciplinary, often including physicians, NPs, RNs, counsellors, dietitians, and outreach workers
Nurse Practitioner (NP)-Led Clinic	1	A solo NP-run clinic in a rural/remote setting with limited access to physicians. The NP provides comprehensive primary care, minor procedures, chronic disease management, and community-based follow-up. The population includes low-income rural residents and older adults. Collaboration with nearby hospital services is essential due to lack of onsite support
Group Practice (Northern)	1	A small group family physician-led clinic located in a remote northern community. The practice serves rural and Indigenous population with limited primary care access. Team includes 5–7 physicians, rotating locums, and administrative staff. Patients often face geographic barriers, chronic disease burden, and cultural care needs. Coordination with visiting specialists and regional services is a key feature

The PaRIS Project was conducted in partnership with the Saskatchewan Ministry of Health, Saskatchewan Medical Association (SMA), Saskatchewan Health Authority (SHA), Saskatchewan Health Quality Council (HQC), Saskatchewan Centre for Patient-Oriented Research (ScPOR), and the Departments of Family Medicine (DFM) and Community Health and Epidemiology (CH&E) at the University of Saskatchewan.

### Participants

Participants in this evaluation included:

- *PCPs:* Family physicians and nurse practitioners who participated in and drove recruitment of the survey implementation.
- *Clinic Administrators and MOAs:* Staff from partnering clinics who were actively involved in supporting the PaRIS survey dissemination and dashboard use.
- *PWLE:* Patients and patient-partners who contributed perspectives on the relevance and interpretation of the dashboards.

### Intervention

**Fig. 1:**
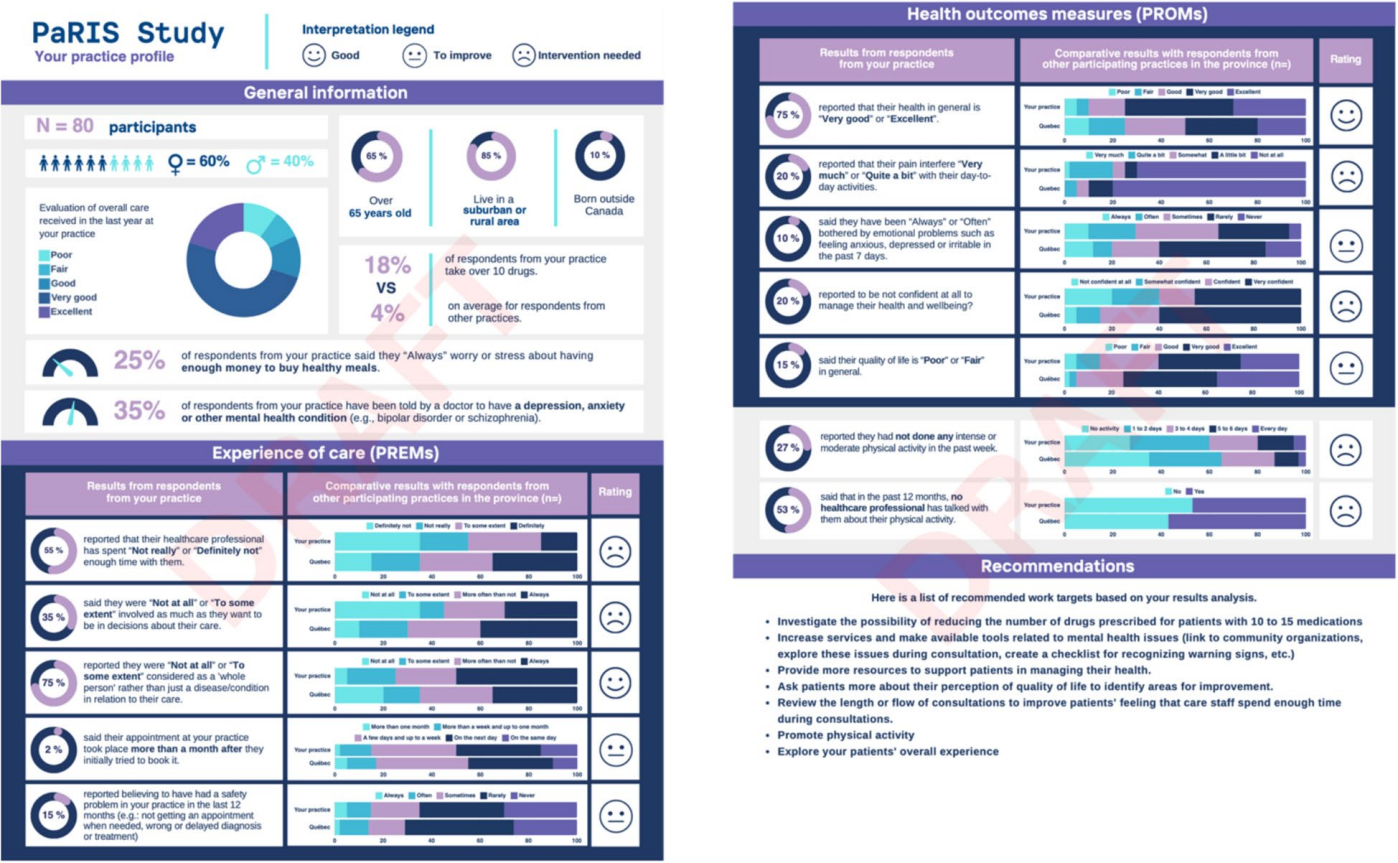
Example OECD PaRIS dashboard showing clinic-level PREM and PROM indicators with comparative benchmarks and interpretive ratings

Our core intervention evaluated was the deployment and clinic-level engagement with the OECD PaRIS dashboards (see sample of data presentation dashboards in Fig. 1.), which displayed aggregated survey findings on patient experiences and outcomes. These dashboards were developed in collaboration with our international and national partners and returned to each participating clinic as part of the data feedback and knowledge translation processes. The dashboards presented clinic-level results alongside comparative benchmarks from other participating practices and provincial averages. They included PREMs such as time spent with providers, involvement in decision-making, being treated as a whole person, access to care (e.g., wait times), and perceived safety issues within care. They also included PROMs such as self-rated health, pain interference with daily activities, mental health (e.g., anxiety or depression), confidence in managing one’s health, quality of life, physical activity levels, and preventive care discussions (e.g., counselling on physical activity). Visualizations combined summary percentages, distribution charts, and comparative bar graphs contrasting clinic-level results with provincial benchmarks. These were accompanied by simple interpretive indicators (e.g., “good,” “to improve,” “intervention needed”) and embedded recommendations to support rapid understanding, reflection, and identification of potential areas for quality improvement at the clinic level. See Fig. 1.

### Participatory evaluation framework

Our evaluation framework was co-developed by and with the members of the research team. Our objective was to learn, discuss and examine with our partners what worked, what didn’t work and what was essential to informing future primary care research within the local context of each clinic. Key measures discussed in the participatory evaluation included:

- Response rates (PCPs and PWLE).
- Perceptions of dashboard relevance.
- Ease and relevance of use and interpretation.
- Identified data-to-practice linkages.
- Recommendations for future engagement and data presentation/visualization strategies.

### Data collection and measures

Data were collected using multiple sources to support both implementation and evaluation:

- Structured and semi-structured feedback sessions (qualitative) conducted with clinic partners during dashboard dissemination; and
- Discussions and reflexive notes (qualitative), which captured reflections on dashboard utility, challenges in interpretation, and suggestions for improvement.

The qualitative data generated through feedback sessions, discussions, and reflexive notes constituted the *primary analytic dataset* for this evaluation.

### Data analysis

This present study did not involve analysis of the underlying OECD PaRIS dataset. Rather, the local PaRIS data informed the development of clinic-level dashboards, which served as the basis for participant engagement and the qualitative evaluation of their interpretation and use.

For the data collected, we coded the qualitative data after transcription following Braun and Clarke’s [26] process for inductive coding, which included the following: familiarizing ourselves with the data, generating initial codes, searching for themes, reviewing themes, defining and naming themes, and producing the final report. This iterative process allowed themes to emerge directly from the data and was conducted collaboratively with input from members of the research team to ensure alignment with the participatory evaluation framework. Themes were co-developed using an interpretive descriptive approach [27], with input from the participants and were grounded in the project’s commitment to relational equity [20]. Challenges, facilitators, and areas for system improvement were triangulated both within and across the various clinical settings.

### Techniques to enhance rigour and trustworthiness

Various techniques to enhance the rigour and trustworthiness of this work were undertaken following the guidelines set forth by Creswell [28], Guba & Lincoln [29], Shenton [30], and Patton [31]. Credibility was supported through member checking; wherein preliminary interpretations were shared with clinic partners and PWLE for validation and refinement. Confirmability was strengthened through collaborative coding, team-based analysis, and triangulation of perspectives across researchers with clinical, engagement, and academic expertise. The lead facilitator (UO) utilized reflexive journaling and regular team debriefings to examine assumptions and minimize researcher bias. Transferability was supported through a detailed description of the study context, participants, and procedures. We followed the SQUIRE 2.0 (Standards for Quality Improvement Reporting Excellence) Guidelines for reporting [32], along with the GRIPP2 (Guidance for Reporting Involvement of Patients and the Public) short form [33], to ensure transparent, systematic, and comprehensive reporting aligned with best practices for quality improvement and participatory research (see Supplementary Files B and C). In reporting the qualitative components of this evaluation, we were guided by principles from the Standards for Reporting Qualitative Research (SRQR) to enhance transparency in data collection and analysis [34].

### Researcher characteristics and reflexivity

Our evaluation was conducted by an interdisciplinary team with expertise in primary care, participatory research, patient(people) engagement, and health services research. The lead author (UO), a participatory researcher, facilitated the evaluation, with oversight from VRR, also experienced in participatory and patient-oriented primary care research. Relational equity was operationalised through the team’s composition and processes. BA, an engagement specialist, and PWLE, facilitated trust-building sessions, while clinicians (ML, JK, AT, KF), researchers (RV, NM) and system administrators (DM, LC, PL), embedded in our partnering clinics contributed contextual insight, supported recruitment, and co-interpreted findings.

Consistent with Participatory Empowerment Evaluation (PEE) principles [18], our evaluation engaged all our participants as partners rather than passive subjects. Clinic (unit) leaders, practitioners, staff, and PWLE were actively involved in sense-making, interpretation, and refinement of findings (in several sessions of this work), which informed both the evaluation outcomes and knowledge translation; thus, were all included as co-authors. Their inclusion as co-authors reflects substantive contributions to the evaluation process and aligns with the partnership-based ethos underpinning the study framework.

### Ethics considerations

This study was reviewed by the University of Saskatchewan’s Behavioral Ethics Board and received an Exemption per Article 2.5 of the Tri-Council Policy Statement (TCPS 2–2022): Ethical Conduct for Research Involving Humans [35].

## Findings

We evaluated the participatory processes within the local PaRIS Project with all the key members of *the PaRIS Saskatchewan Research Team* which spanned across the ten clinics that partnered with the Project. This participatory evaluation revealed a dynamic and relationally driven process through which clinics engaged with the OECD PaRIS Project and dashboards. The participating clinics described their experience not as a one-time data-sharing event but as evolving relationships grounded in trust, mutual respect, and co-learning. We described and organized the findings around the main objectives of this evaluation: (1) to describe engagement in and with the OECD PaRIS Project, (2) to identify key facilitators and challenges to engagement and data interpretation, and (3) to explore opportunities for improving the participatory processes in future data collection and application in primary care settings.

### Engagement in and with the PaRIS project and dashboards

Engagement across participating clinics was notably high and driven by trust, transparency, and a sense of shared purpose that had been established during the PaRIS survey rollout. Participants described how early and ongoing engagement (including being involved prior to data collection, opportunities for dialogue during implementation, and follow-up discussions) contributed to building trust and fostering a sense of shared ownership in the process.

Participants described the data presentation dashboards as “validating” and “empowering,” particularly when specific indicators (such as patient-reported experiences of communication, being treated as a whole person, involvement in decision-making, and overall ratings of care) aligned with the care values and relational approaches emphasized within their clinics. One urban-based clinic manager remarked:

*“It’s one thing to feel we’re doing a good job. Seeing it on paper from our patients, that’s powerful…it gives us something concrete to build on.”*

For clinic administrators and managers, these insights were often tied to indicators related to access, coordination, and safety (e.g., wait times, delays in care, and reported safety concerns), which helped identify operational areas for improvement and supported planning for workflow and service delivery changes. These were often interpreted alongside comparative benchmarks, which helped clinics identify whether observed patterns reflected local strengths or broader system-level trends.

For many PCPs, specific elements of the dashboard data affirmed the values already embedded in their care delivery and validated their efforts to build continuity and trust with their patients. In particular, measures related to patients feeling listened to, being treated as whole persons, and reporting positive overall care experiences were seen as reflecting relational aspects of care that clinicians intentionally prioritized. One urban-based PCP commented that:

*“the data reminded us that we are making a difference…it’s not always visible in the day-to-day, but this helped us step back and see it.”*

The sense of co-ownership fostered throughout the project was also a recurring theme. PCPs described feeling respected and included in a way that made them more open to engaging in and with the project. The sense of co-ownership fostered throughout the project was also a recurring theme. PCPs described feeling respected and included in a way that made them more open to engaging in and with the project. This sense of co-ownership and inclusion was closely tied to relational trust, as participants described feeling more accountable to the data and more motivated to engage when they perceived the process as collaborative rather than externally imposed. Being heard, consulted, and engaged throughout the process further reinforced this trust and increased participants’ willingness to meaningfully participate in and act on the data. Another urban-based PCP reflected on how the participatory nature of the process shaped their openness to engaging with the data:

*“Because we were brought in early and not just handed something to interpret, there’s more willingness to reflect and act on what’s there.”*

Across participating clinics, this early involvement appeared to have reframed the way data collection in surveys should be approached–not as top-down process but as an opportunity for reciprocal trust-building, and co-creation from the bottom-up. This overall sense of relational trust, we believe, facilitated data reception and positioned the dashboards as valuable tools for internal continuous quality improvement and patient advocacy.

### Facilitators and challenges in engagement and data interpretation/use

#### Facilitators identified

We identified several factors that helped make the PaRIS data engagement more meaningful. They included:

- The relational and transparent approach used to foster trust and interest.
- Contextualized and individualized data presentation of findings; as well as feedback that incorporated clinic-specific areas for improvement, and
- Clarity of language used in summary narratives, even when some visuals appeared complex or difficult to understand.

Participants noted that narrative summaries were particularly helpful in interpreting indicators related to patient experience and outcomes, especially when paired with comparative data that contextualized clinic performance. A PWLE working with a rural-based clinic noted:

*“It didn’t feel like data was being dropped on us…it felt like an invitation to make meaning together.”*

This was frequently interpreted as a reflection of trust-building, where participants felt their perspectives were not only solicited but genuinely valued within the process. Some of the PWLE participants often connected their experiences to specific indicators within the dashboards, particularly those related to communication, involvement in decision-making, and emotional wellbeing, highlighting how these measures reflected their lived experiences of care and their desire to be meaningfully included in care processes.

Participating clinics also appreciated the feedback loops established throughout the entire project, up to the data dissemination meetings and one-on-one follow-ups. Seven of the ten participating clinics reported that the data affirmed their existing practice goals, particularly through indicators related to communication, continuity of care, and patient involvement in decision-making. These clinics interpreted these indicators as evidence that their relational approaches to care were translating into meaningful patient experiences.

#### Challenges identified

Despite these strengths, participants also noted challenges with the engagement processes and data presentation dashboards, which included the following:

1. *Visual complexity*: Several participants indicated a difficulty in interpreting dense data visualizations either on mobile or other smaller devices, particularly bar graphs and comparative trends. These challenges were particularly pronounced for indicators presented through comparative bar graphs and distribution charts, which some participants found difficult to interpret without facilitation.
  An urban-based clinic administrator shared that:
  “…the bar graphs looked fine in the email, but when we opened them on our phones during huddles, they were hard to explain…especially without someone walking us through.”
  While the narrative summaries included in the data presentation, dashboards were praised for their clarity, some participants felt that a more intuitive design would enhance accessibility.
2. *Temporal barriers to accessing full data for use in other comparative analysis*: Another challenge that emerged was around the timing and release of comparative data (for context, some variables in the provincial data were embargoed by OECD for over a year). Some clinics expressed a desire to see how most of their results aligned with the broader provincial trends but noted that embargoes on certain key variables limited their ability to situate their performance.
  An urban-based PCP reflected:
  “We know we’re doing okay, but without knowing where others are, it’s hard to know where to focus next.”
3. *Time constraints*: Time constraints were another less recurring theme. Although the data were considered meaningful, a few participants (particularly PCPs) noted the difficulty of creating protected time to review and act upon them. A rural-based PCP indicated:
  “This kind of data is useful,...but only if we have the time and space to actually talk about it as a team.”
4. *Limited engagement among certain patient groups*: Some patient groups, especially older adults and those with limited digital literacy in some partnering clinics, reported that they were less likely to engage with or benefit from the formats of the data presentation dashboards. This was especially relevant for indicators related to patient experience and outcomes, which may not have been equally accessible across all patient groups due to format and delivery methods.
  “Some of our older patients aren’t going to engage with dashboards like this…they’re not using computers or smartphones in that way, so we have to think about other ways of sharing this information.”– rural-based clinic administrator
  “I wouldn’t really use something like that…I’m not very comfortable with computers, and it just feels like too much to take in.”– PWLE

### Opportunities for improvement and future engagement

Despite the challenges identified, participants across partnering clinics described how the participatory nature of the PaRIS Project in Saskatchewan fostered an environment of learning and forward-thinking. Building on this, they offered several practical suggestions to strengthen future participatory engagement processes and enhance the usability of data outputs.

A consistent theme that emerged from our conversations was the need to simplify data visualizations and improve accessibility, particularly for teams working in fast-paced or resource-constrained settings. As one urban-based PCP noted,

*“The dashboard is helpful, but it’s a lot to take in…we need something simpler that we can quickly interpret during a busy clinic day.”*

Participants also emphasized the importance of optimizing presentation formats for mobile or low-bandwidth environments, particularly for rural and remote settings.

*“If it’s not easy to pull up quickly, especially in places with limited connectivity, it’s just not going to get used in day-to-day practice.” – urban-based PCP*

Participants further highlighted opportunities to enhance real-time engagement and data collection. Several suggested integrating feedback mechanisms directly into clinical workflows, such as tablets or in waiting rooms. As one urban-based clinic staff member explained,

*“If patients could give feedback right there while they’re waiting, it would make the process easier and more continuous.”*

Another key recommendation was the need to tailor engagement and dissemination strategies for diverse populations, including multilingual and digitally underserved groups. Participants noted that current approaches may not fully capture the experiences of all patients. One urban-based clinic manager reflected,

*“Not everyone we serve can easily engage with these tools…there needs to be other ways of reaching people.”*

Notably, several participants serving rural and remote areas stressed the need to extend engagement timelines, particularly when working with Indigenous communities, to ensure that processes were culturally appropriate and grounded in trust-building. One urban-based Clinical Administrator noted:

*“…early engagement is key as these relationships take time…you can’t rush community engagement, especially in communities that have been over-researched as we see here.”*

Overall, there was a palpable sense of optimism across our partnering clinics about what this participatory evaluation had begun to build. Participants emphasized the importance of sustaining relational approaches in future iterations:

*“What made this different is that it didn’t feel extractive. We were part of it. Keep doing that, and more people will join.”– PWLE*

## Discussion

The participatory evaluation of the OECD PaRIS Project in Saskatchewan demonstrated that the success of healthcare data initiatives depends not only on technical excellence but on the strength of relationships nurtured over time. While the dashboards themselves were tools of knowledge translation, we truly believe that it was the relational fabric woven throughout the participatory processes that truly brought them to life [16, 17].

Across all ten clinics, it became clear that the usual engagement in PREMS and PROMS was not transactional but deeply relational. Participants repeatedly emphasized that their willingness to participate, jointly interpret, and apply the data was grounded in the trust that had been cultivated before the data dashboards ever arrived. This trust was earned through early and continuous authentic engagement, responsiveness to feedback, and a shared commitment to honoring their diverse voices, particularly those of people with lived experience (PWLE). It was all about these relationships.

In interpreting our results, it is important to situate them in the broader literature on what drives patient participation in PROMs/PREMs. Studies [36–40], suggest that enhanced participation in PREMs/PROMs was shaped by multiple, interrelated factors including features of the instrument itself (e.g. length, clarity, ease of use), individual beliefs and motivations, and organizational/relationship-level facilitators (e.g. clinician engagement, workflow integration, communication). For example, McCabe et al. [38] identified five key domains influencing PROM/PREM implementation to include: (1) characteristics of the measures (e.g. burden, comprehensibility), (2) beliefs of individuals about utility and burden, (3) how the instruments are administered, (4) embedding them within clinical workflows, and (5) incentives or supports for use. Al-Antary and Associates [37] showed how occasional reminders, mixed modes of administration, patient education, and clinician involvement were effective to increasing response rates. Xu et al. [40] emphasized that usability, intuitive platform design, and minimal effort were central to fostering participant engagement in electronic surveys. Sand-Svartrud et al. [39] showed that a shorter (“brief”) PROMs set significantly increased completeness relative to longer ones, and, Vilkki et al. [36] reported factors such as questionnaire length, patient age, and health behaviors were linked with high response likelihood. These studies suggest that there are no single levers to enhancing participation in PREMs/PROMs, but it was more likely when the burden was low, the value was manifest, and organizational supports were strong.

While these cited studies hold contextual merits, we believe, in our case, that the relationships (between patients and clinicians/research staff) acted as cross-cutting facilitators (e.g. patients felt more comfortable asking clarifying questions), boosting intrinsic motivation (e.g., patients felt their voices “mattered” to the clinician), and enhanced follow-through (e.g., clinicians personally reminding or encouraging). From our evaluation, we identified instances where clinicians or care coordinators directly engaged participants (e.g. via follow-up calls or personalized messages) which aligned with higher completion rates, consistent with our relational leverage.

The importance of relationships in shaping meaningful outcomes in primary care is echoed within both the literature and the policies from the 1978 Alma-Ata Declaration [41], to the 1986 Ottawa Charter for Health Promotion [42], and to the 2018 Astana Declaration [43]. We defined relationships in this context, as equitable, dynamic, reciprocal connections between individuals, communities, and systems, built on mutual respect, trust, and shared goals [19]. Relationships in this sense, were not merely transactional or hierarchical; rather they were horizontal, collaborative and transformative, fostering a sense of belonging, ownership, and shared responsibility in its co-creative processes. With Indigenous communities, relationships are considered a *sine qua non* for enhancing reconciliatory efforts by and with them [44, 45], as it ensures that their voices and needs are actively integrated into the research processes [17]. Developing relationship-driven or focused research initiatives fosters trust, continuity, and collaboration, which are essential for patient-centered care and meaningful research outcomes [46, 47]. In primary care or other health research, relationships drive bottom-up processes, empowering communities to co-create processes and systems that are more responsive to their unique needs. Relationships as such, enhances equity, shared decision-making, and long-term sustainability in both research and evaluation processes [45, 47]. The lessons from our participatory evaluation reaffirmed these.

We believe that the feeling of co-ownership was a hallmark of the enhanced participatory approach we adopted within this project. The participatory framework we used which was grounded in relational equity and mutual respect, created a space where clinics could engage deeply not just with the PaRIS data, but with one another [19]. We believe many participants saw the spaces we created as an opportunity to bring their values into alignment with data and decision-making, as well as to lay the foundation for future quality improvement efforts. Our evaluation findings and lessons learned also aligned with the growing recognition in healthcare regarding data stewardship. Data stewardship is inseparable from relational stewardship [48, 49]. If patients are to see their feedback as more than data points, or if PCPs are to use such data for real change, the processes must feel meaningful, transparent and reciprocal [46, 50, 51]. In contexts within Indigenous health, for instance, this means honoring principles of Ownership, Control, Access and Possession (OCAP®) and creating extended timelines for relationship-building before introducing metrics or research questions [46, 51, 52]. Similarly, in digitally underserved populations, this could mean re-imagining the data collection tools altogether (e.g., offering simpler visuals, multilingual resources, and in-person walkthroughs to bridge interpretation gaps) [50].

Our participatory evaluation affirms that building trust-based relationships in PREMs and PROMs data collection processes could be a viable mechanism through which data becomes relational because people engage when their voices are seen to matter, where participation in data collection is meaningful and when system transformation begins. Using a participatory framework grounded in relational equity, we were able to surface not only how partners engaged with the PaRIS Project processes, but also why that engagement mattered. Our partners did not simply interpret their data; they co-owned it, used it to reflect on their own practices, and envision paths forward.

## Conclusion

This participatory evaluation of the OECD PaRIS Project in Saskatchewan explored how relational approaches shaped partner engagement with patient-reported outcomes and experience data. Our evaluation findings showed that early, inclusive, and trust-based engagement led to strong clinic participation, a sense of co-ownership of the data, and more willingness to use the findings for internal reflection and continuous quality improvement. These findings affirmed that participation was most meaningful when people felt their voices were respected and their feedback utilized. Future directions of this work will build on the lessons learned from this evaluation to continue with co-creating more inclusive, culturally responsive, and sustainable approaches for engaging both patients, practitioners and clinic managers in primary care data initiatives to inform meaningful system change.

## Supplementary Information

Supplementary Material 1.

## Data Availability

The data that support the findings of this study are available from the authors upon reasonable request.

## Abbreviations

CH&E: Community Health and Epidemiology
DFM: Department of Family Medicine
HQC: Health Quality Council
MOA: Medical Office Assistant
OECD: Organization for Economic Co-operation and Development
PaRIS: Patient-Reported Indicator Surveys
PCP: Primary Care Practitioner
PREMs: Patient-Reported Experience Measures
PROMs: Patient-Reported Outcome Measures
PWLE: People with Lived Experience
SCPOR: Saskatchewan Centre for Patient-Oriented Research
SHA: Saskatchewan Health Authority
SMA: Saskatchewan Medical Association
SQUIRE: Standards for Quality Improvement Reporting Excellence
