# Supplementary material for: Evaluating the participatory processes within the OECD PaRIS project in Saskatchewan: lessons learned about recruitment and engagement
Source: BMC Prim Care. 2026 Apr 1;27:189. doi: 10.1186/s12875-026-03288-4 (PMC13169619; doi:10.1186/s12875-026-03288-4)
Supplement: Supplementary file 1 — Supplementary Material 1. [file 12875_2026_3288_MOESM1_ESM.docx]

**Supplementary File A: PaRIS Patient Survey**


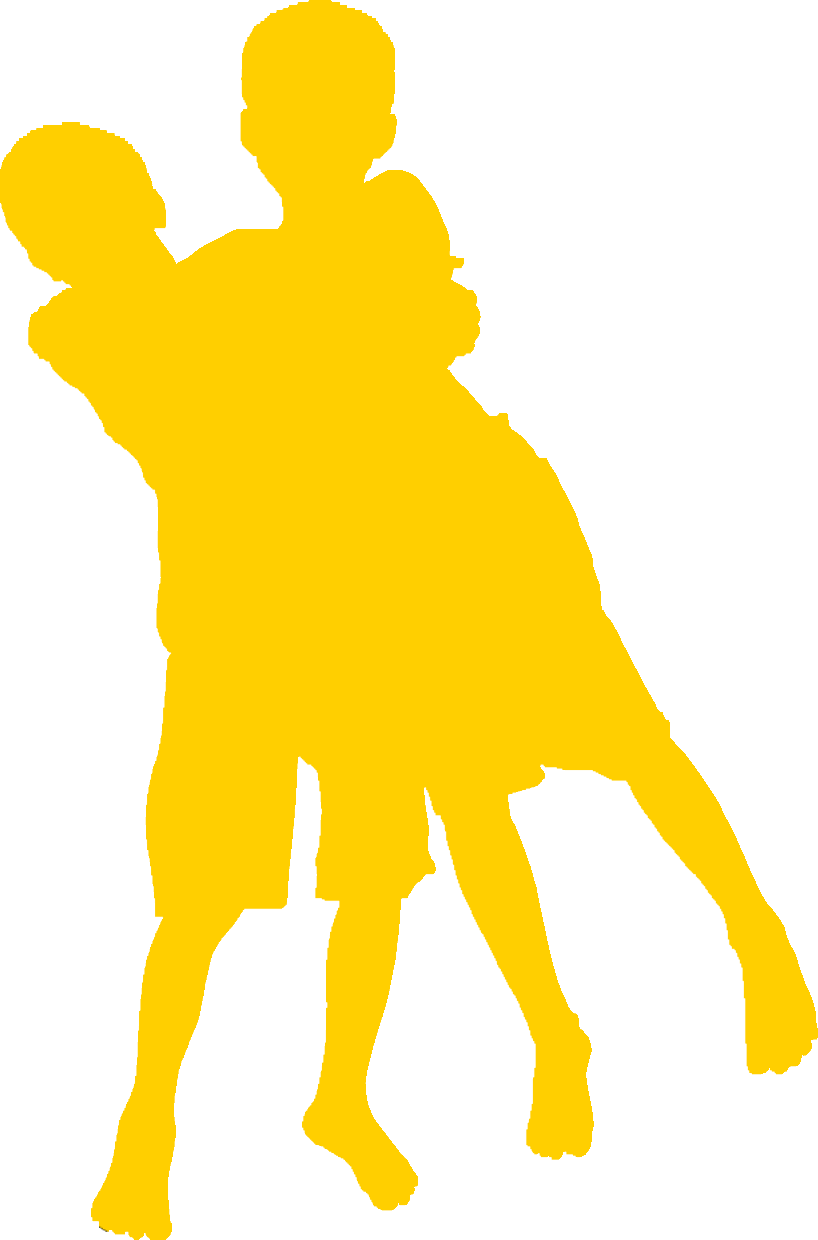

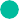

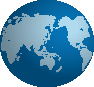


Patient Reported Indicator Surveys

PaRIS Patient Questionnaire (PaRIS-PQ)


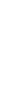

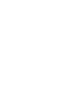


# OECD PaRIS Patient Questionnaire (PaRIS-PQ)

Version for scripting online survey 2024

© OECD 2024 The use of this work, whether digital or print, is governed by the Terms and Conditions to be found at <https://www.oecd.org/termsandconditions>.

**
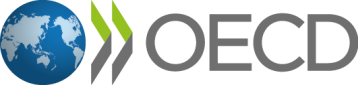
**

Table of contents

[Background 3](#_bookmark0)

[OECD PaRIS-PQ for scripting 4](#_bookmark1)

[Annex A. Source questionnaires of the PaRIS-PQ 41](#_bookmark2)

The OECD's Patient-Reported Indicator Surveys (PaRIS) initiative aims to evaluate healthcare outcomes and patient experiences internationally, with a focus on primary care performance. The primary tool used in this initiative is the PaRIS Patient Questionnaire (PaRIS-PQ), designed to assess the experiences and outcomes of patients aged 45 years and older managed in primary care. Alongside patient-reported outcomes and experience measures (PROMs and PREMs), the PaRIS-PQ includes items about sociodemographic factors, health behaviours, and healthcare capabilities. To offer further context and insights into patient-reported experiences and outcomes, a complementary questionnaire called the [PaRIS Primary Care Practice Questionnaire](https://www.oecd.org/health/paris/PaRIS-provider-questionnaire.pdf) (PaRIS-PCPQ) is employed to evaluate practice characteristics and the care delivered, particularly focusing on chronic condition management.

The development of the PaRIS-PQ involved close collaboration with policymakers, patients, and primary care providers. It is grounded in the PaRIS conceptual framework, developed through a rigorous process involving literature review and a modified Delphi process. The questionnaire underwent translation into the languages of participating countries, cross-national cognitive testing, and pilot testing in a field trial.

This document presents the English source version of the PaRIS-PQ for scripting an online survey, used for data collection between 2022 and 2023. Additionally, it includes the sources for the items incorporated into the PaRIS-PQ.

The [PaRIS Initiative](http://www.oecd.org/health/paris/) (Patient-Reported Indicator Surveys) of the OECD aims to develop and implement internationally comparable patient-reported indicators, more specifically patient-reported outcome measures (PROMs) and patient-reported experience measures (PREMs).

The OECD developed the PaRIS survey instruments with an international consortium of Nivel, Ipsos, University of Exeter, Optimedis AG and Avedis Donabedian Research Institute.

**Note to the reader:** The PaRIS PQ was administered mainly online, but also on paper and by telephone depending on the best suited mode for the country. The layout of this document uses the PaRIS PQ version for scripting the online survey, rather than the paper-based version. Please note following elements for clarification:

- ASK ALL: item is applicable to all respondents
- ASK IF: applicability of the item is conditional on another item
- (SINGLE): items where respondents can give one of the response options
- (MULTI): items where respondents may tick multiple response options
- (LOOP): items consisting of multiple questions, each with the same answering categories

This survey is being carried out by <<insert national relevant organization in each country>> in collaboration with the Organisation for Economic Cooperation and Development (OECD). <<insert country>> is taking part in an international survey to improve health services locally and nationally.

The survey asks questions about your experience of care and treatment by your

**<<primary care provider>>. *Primary care services are usually the first point of contact with the health service for a wide range of new and long-term health problems. They are frequently delivered by teams of health care professionals, usually coordinated by a family doctor or a general practitioner working in a primary care centre. In your case this may be also another member of the primary care team, such as a nurse, or another specialist doctor.***

In addition, there are some questions about you and your health. Your answers are important and will help policy makers and health care providers improve health care based on patients’ needs.

The survey should take around 25 minutes, depending on the answers you give. If you cannot complete the survey in one session and would like to come back and finish later, just close the window and your responses will be saved. When you are ready, you can return to the same point.

Taking part in this survey is voluntary and your answers will be treated in confidence. If you do not wish to answer a question, please click the >> button twice to move on.

For more information about this survey and how we will use your data, please click on the link below to view the privacy policy.

If you have any queries about the questionnaire, please call our helpline number on [LOCAL NUMBER] or email [[LOCAL EMAIL].](mailto:CQCsurveys@ipsos.com)

CONSENT (SINGLE CODE)

Do you agree to take part in the survey?

**Yes** CONTINUE

**No** CLOSE

SECTION 1 “Your Health” TIMING START

ASK ALL NEW PAGE

### Your health

The first few questions are about your health.

| **Questions** | | | | **Source** |
| --- | --- | --- | --- | --- |
| Q_AGE (SINGLE)  **How old are you?** | | | | Modified from OECD PISA-  D Main Study Teacher Questionnaire IS |
|  | 1 | 44 years old or younger |  |  |
|  | 2 | 45-49 years old |  |  |
|  | 3 | 50-54 years old |  |  |
|  | 4 | 55-59 years old |  |  |
|  | 5 | 60-64 years old |  |  |
|  | 6 | 65-69 years old |  |  |
|  | 7 | 70-74 years old |  |  |
|  | 8 | 75-79 years old |  |  |
|  | 9 | 80-84 years old |  |  |
|  | 10 | 85 years or older |  |  |
|  | 97 | Prefer not to say |  |  |

IF Q_AGE = 1 PARTICIPANTS ARE INELIGIBLE AND SHOULD NOT CONTINUE ASK ALL

NEW PAGE

|  |  |
| --- | --- |
|  |  |
|  |  |
|  |  |
|  |  |
|  |  |

| **Questions** | | | | **Source** |
| --- | --- | --- | --- | --- |
| Q_GLOBAL (LOOP) | | | | PROMIS® |
| GLOBAL01 **In general, would you say your health is:** | | | | Scale v1.2 – |
| GLOBAL02 **In general, would you say your quality of life is:** | | | | Global Health |
| GLOBAL03 **In general, how would you rate your physical health?** | | | |  |
| GLOBAL04 **In general, how would you rate your mental health,** | | | |  |
| **including your mood and your ability to think?** | | | |  |
| GLOBAL05 **In general, how would you rate your satisfaction with** | | | |  |
| **your social activities and relationships?** | | | |  |
| GLOBAL9R **In general, please rate how well you carry out your** | | | |  |
| **usual social activities and roles. (This includes** | | | |  |
| **activities at home, at work and in your community, and** | | | |  |
| **responsibilities as a parent, child, spouse, employee,** | | | |  |
| **friend, etc.)** | | | |  |
| Responses (SINGLE) | | | |  |
|  | 1 | Excellent |  |  |
|  | 2 | Very good |  |  |
|  | 3 | Good |  |  |
|  | 4 | Fair |  |  |
|  | 5 | Poor |  |  |

ASK ALL NEW PAGE

| **Questions** | | | | **Source** |
| --- | --- | --- | --- | --- |
| Q_GLOBAL06 (SINGLE)  **To what extent are you able to carry out your everyday physical activities such as walking, climbing stairs, carrying groceries, or moving a chair?** | | | | PROMIS®  Scale v1.2 – Global Health |
|  | 1 | Completely |  |  |
|  | 2 | Mostly |  |  |
|  | 3 | Moderately |  |  |
|  | 4 | A little |  |  |
|  | 5 | Not at all |  |  |

ASK ALL NEW PAGE

The next few questions are about any specific symptoms you may have recently experienced.

ASK ALL NEW PAGE

| **Questions** | | | | **Source** |
| --- | --- | --- | --- | --- |
| Q_PROMISDYSCB001 (SINGLE)  **In the past 7 days**  **I have been short of breath** | | | | PROMIS Item Bank v. 1.0 – Dyspnea |
|  | 1 | Not at all |  |  |
|  | 2 | A little bit |  |  |
|  | 3 | Somewhat |  |  |
|  | 4 | Quite a bit |  |  |
|  | 5 | Very much |  |  |

ASK ALL NEW PAGE

| **Questions** | | | | **Source** |
| --- | --- | --- | --- | --- |
| Q_GLOBAL08R (SINGLE)  **In the past 7 days**  **How would you rate your fatigue on average?** | | | | PROMIS®  Scale v1.2 – Global Health |
|  | 1 | None |  |  |
|  | 2 | Mild |  |  |
|  | 3 | Moderate |  |  |
|  | 4 | Severe |  |  |
|  | 5 | Very severe |  |  |

ASK ALL NEW PAGE

| **Questions** | | | | **Source** |
| --- | --- | --- | --- | --- |
| Q_PROMISPAIN (SINGLE)  **In the past 7 days**  **How much did pain interfere with your day-to-day activities?** | | | | PROMIS Adult Short Form v1.0 - Pain Interference |
|  | 1 | Not at all |  |  |
|  | 2 | A little bit |  |  |
|  | 3 | Somewhat |  |  |
|  | 4 | Quite a bit |  |  |
|  | 5 | Very much |  |  |

ASK ALL NEW PAGE

| **Questions** | | | **Source** |
| --- | --- | --- | --- |
| Q_GLOBAL07R (SINGLE)  **In the past 7 days**  **How would you rate your pain on average?** | | | PROMIS®  Scale v1.2 – Global Health |
|  | 0-No pain |  |  |
|  | 1 |  |  |
|  | 2 |  |  |
|  | 3 |  |  |
|  | 4 |  |  |
|  | 5 |  |  |
|  | 6 |  |  |
|  | 7 |  |  |
|  | 8 |  |  |
|  | 9 |  |  |
|  | 10-Worst imaginable pain |  |  |

ASK ALL NEW PAGE

| **Questions** | | | | **Source** |
| --- | --- | --- | --- | --- |
| Q_ GLOBAL10R (SINGLE)  **In the past 7 days**  **How often have you been bothered by emotional problems such as feeling anxious, depressed or irritable?** | | | | PROMIS®  Scale v1.2 – Global Health |
|  | 1 | Never |  |  |
|  | 2 | Rarely |  |  |
|  | 3 | Sometimes |  |  |
|  | 4 | Often |  |  |
|  | 5 | Always |  |  |

ASK ALL NEW PAGE

|  |  |
| --- | --- |
|  |  |
|  |  |
|  |  |
|  |  |

| **Questions** | **Source** |
| --- | --- |
| **(REPEAT ON EACH SCREEN) For the next few questions, please think about how you have been feeling over the last two weeks.**  QWHO5 (LOOP)   1. **I have felt cheerful and in good spirits** 2. **I have felt calm and relaxed** 3. **I have felt active and vigorous** 4. **My daily life has been filled with things that interest me** 5. **I woke up feeling fresh and rested**   Responses (SINGLE)   - 1. All of the time   2. Most of the time   3. More than half of the time   4. Less than half of the time   5. Some of the time   6. At no time | WHO Well-being Index (WHO-5) |

SECTION 1 ‘Your health’ TIMING END

|  |  |
| --- | --- |
|  |  |
|  |  |
|  |  |
|  |  |
|  |  |

SECTION 2 ‘Managing your health and health care’ TIMING START

ASK ALL NEW PAGE

### Managing your health and health care

The next few questions ask about your recent physical activity, diet and use of tobacco and alcohol and the support you may have received from health care professionals (doctors, nurses, and other health care professionals). Please respond in relation to your experience of care and treatment by your <<primary care provider>>*.* Primary care services are usually the first point of contact with the health service for a wide range of new and long-term health problems.

ASK ALL

NEW PAGE (SAME SCREEN)

| **Questions** | | | | **Source** |
| --- | --- | --- | --- | --- |
| Q_EXERCISE (SINGLE)  **In the past week, on how many days did you do at least 30 minutes of either vigorous or moderate activity (such as running, swimming, riding a bike, brisk walking, or dancing)? Vigorous physical activities typically make you breathe much harder than normal, whereas moderate activities make you breathe somewhat harder than normal.** | | | | Modified from IPAQ-SF |
|  | 1 | Every day |  |  |
|  | 2 | 5 to 6 days |  |  |
|  | 3 | 3 to 4 days |  |  |
|  | 4 | 1 to 2 days |  |  |
|  | 5 | I did not do any vigorous or moderate activity in the past week |  |  |
|  | 6 | Not sure |  |  |

ASK ALL NEW PAGE

| **Questions** | | | | **Source** |
| --- | --- | --- | --- | --- |
| Q_ADVPHYSACT (SINGLE)  **In the past 12 months, has any health care professional talked with you about your physical activity?** | | | | Modified from CWF2017IHP |
|  | 1 | Yes |  |  |
|  | 2 | No |  |  |
|  | 3 | Not sure |  |  |

ASK ALL NEW PAGE

| **Questions** | | | | **Source** |
| --- | --- | --- | --- | --- |
| Q_EHISFR (SINGLE)  **How often do you eat fruit, excluding juice squeezed from fresh fruit or made from concentrate?** | | | | Modified from European Health Interview 2020 Edition |
|  | 1 | More than once a day |  |  |
|  | 2 | Once a day |  |  |
|  | 3 | 4 to 6 days a week |  |  |
|  | 4 | 1 to 3 days a week |  |  |
|  | 5 | Less than once a week |  |  |
|  | 6 | Never |  |  |

ASK ALL NEW PAGE

| **Questions** | | | | **Source** |
| --- | --- | --- | --- | --- |
| Q_EHISVG (SINGLE)  **How often do you eat vegetables or salad, excluding potatoes and fresh juice or juice made from concentrate?** | | | | Modified from European Health Interview 2020 Edition |
|  | 1 | More than once a day |  |  |
|  | 2 | Once a day |  |  |
|  | 3 | 4 to 6 days a week |  |  |
|  | 4 | 1 to 3 days a week |  |  |
|  | 5 | Less than once a week |  |  |
|  | 6 | Never |  |  |

ASK ALL NEW PAGE

| **Questions** | | | | **Source** |
| --- | --- | --- | --- | --- |
| Q_ ADVDIET (SINGLE)  **In the past 12 months, has any health care professional talked with you about healthy eating?** | | | | Modified from CWF2017IHP |
|  | 1 | Yes |  |  |
|  | 2 | No |  |  |
|  | 3 | Not sure |  |  |

ASK ALL NEW PAGE

| **Questions** | | | | **Source** |
| --- | --- | --- | --- | --- |
| Q_EHISSK01 (SINGLE)  **Do you smoke any tobacco products (excluding electronic cigarettes or similar electronic devices)?** | | | | European Health Interview 2020 Edition |
|  | 1 | Yes, daily |  |  |
|  | 2 | Yes, occasionally |  |  |
|  | 3 | Not at all |  |  |

ASK IF Q_EHISSK01 = 3 NEW PAGE

| **Questions** | | | | **Source** |
| --- | --- | --- | --- | --- |
| Q_EHISSK04 (SINGLE)  **Have you ever smoked tobacco products (excluding electronic cigarettes or similar electronic devices) daily, or almost daily, for at least one year?** | | | | Modified from European Health Interview 2020 Edition |
|  | 1 | Yes |  |  |
|  | 2 | No |  |  |

ASK IF Q_EHISSK01 = 1 or 2 OR Q_EHISSK04=1 NEW PAGE

| **Questions** | | | | **Source** |
| --- | --- | --- | --- | --- |
| Q_CWF2017IHP (SINGLE)  **In the past 12 months, has any health care professional talked with you about the health risks of smoking or using tobacco and ways to quit?** | | | | Modified from CWF2017IHP |
|  | 1 | Yes |  |  |
|  | 2 | No |  |  |
|  | 3 | Not sure |  |  |

ASK ALL NEW PAGE

| **Questions** | | | | **Source** |
| --- | --- | --- | --- | --- |
| Q_EHIS (SINGLE)  **In the past 12 months, how often have you had an alcoholic drink of any kind (beer, wine, cider, spirits, cocktails, premixes, liquor, homemade alcohol …)?** | | | | European Health Interview 2020 Edition |
|  | **1** | Every day or almost |  |  |
|  | **2** | 5-6 days a week |  |  |
|  | **3** | 3-4 days a week |  |  |
|  | **4** | 1-2 days a week |  |  |
|  | **5** | 2-3 days in a month |  |  |
|  | **6** | Once a month |  |  |
|  | **7** | Less than once a month |  |  |
|  | **8** | Not in the past 12 months, as I no longer drink alcohol |  |  |
|  | **9** | Never, or only a few sips or tries, in my whole life |  |  |

ASK IF Q_EHIS = 1 TO 8 NEW PAGE

| **Questions** | | | | **Source** |
| --- | --- | --- | --- | --- |
| Q_CWF2017IHP (SINGLE)  **In the past 12 months, has any health care professional talked with you about alcohol use?** | | | | Modified from CWF2017IHP |
|  | 1 | Yes |  |  |
|  | 2 | No |  |  |
|  | 3 | Not sure |  |  |

SECTION 2 ‘Managing your health and health care’ TIMING END

SECTION 3 ‘PN and CONFIDENT’ TIMING START

ASK ALL NEW PAGE

Q_PN (show each question on separate screens)

(Put this text on separate screen). The next few questions are about how you and the health care professionals that you usually see support your health and health care. Please respond in relation to your experience of care and treatment by your <<primary care provider>>.

|  | **Questions** | **Source** |
| --- | --- | --- |
| PN1 | **I rely on health care professionals to tell me everything I need to know to manage my health** | Modified from Porter-Novelli Consumer Preferences Scale |
| PN2 | **Most health issues are too complex for me to understand** | Porter-Novelli Consumer Preferences  Scale |
| PN3 | **I actively try to prevent diseases and illnesses** | Porter-Novelli  Consumer Preferences Scale |
| PN4 | **I leave it to health care professionals to make the right decisions about my health** | Modified from Porter-Novelli Consumer  Preferences Scale |
| PN5 | **It is important to me to be informed about health issues** | Porter-Novelli  Consumer Preferences Scale |
| PN6 | **I need to know about health issues so I can keep myself and my family healthy** | Porter-Novelli Consumer Preferences Scale |
| PN7 | **I have difficulty understanding a lot of the health information that I read** | Porter-Novelli Consumer Preferences  Scale |
| PN8 | **My health care professionals and I work together to manage my health** | Modified from Porter-  Novelli Consumer Preferences Scale |
| PN9 | **When I read or hear something that is relevant to my health care, I bring it up with my health care**  **professionals** | Modified from Porter-Novelli Consumer  Preferences Scale |
| PN10 | **I try to understand my personal health risks** | Porter-Novelli  Consumer Preferences Scale |
| PREOS2m | **When I think something is wrong with my health care, I raise my concerns with my health care professionals** | Modified from PREOS-PC-6 |

Responses (SINGLE)

| 1 | Strongly disagree |
| --- | --- |
| 2 | Disagree |
| 3 | Neither agree nor disagree |
| 4 | Agree |
| 5 | Strongly agree |

ASK ALL NEW PAGE

Q_CONFIDENT (LOOP)

|  | **Questions** | **Source** |
| --- | --- | --- |
| P3CEQ10 | **How confident are you that you can manage your own health and wellbeing?** | Modified from Person-Centred Coordinated Care Experience Questionnaire  (P3CEQ) |
| MPBSPAINSTRC | **How confident are you that you can follow instructions from health care professionals about how you should care for yourself at home?** | Modified from Medicare Patient Engagement  Questions |
| MPBSPAMEDREC | **How confident are you that you can follow instructions from health care professionals about how to change your habits or lifestyle?** | Modified from Medicare Patient  Engagement Questions |
| MPBSPANECESS | **How confident are you that you can identify when it is necessary for you to get medical care?** | Modified from Medicare Patient  Engagement Questions |
| MPBSPASIDEFX | **How confident are you that you can identify when you are having side effects from your medications?** | Modified from Medicare Patient Engagement  Questions |
| eHEALS | **How confident are you in using information from the Internet to make health decisions?** | Modified from eHealth Literacy  Scale (eHEALS) |

Responses (SINGLE)

| 1 | Very confident |
| --- | --- |
| 2 | Confident |
| 3 | Somewhat confident |
| 4 | Not confident at all |
| 5 | Does not apply (MPBSPASIDEFX and eHEALS only) |

SECTION 3 ‘PN and CONFIDENT’ TIMING END

SECTION 4 ‘Your experience of health care – part 1’ TIMING START

ASK ALL NEW PAGE

### Your experience of health care

Please respond in relation to your experience of care and treatment by your <<primary care provider>>.

ASK ALL NEW PAGE

Q_CWF1130 (SINGLE)

| **Questions** | | | | **Source** |
| --- | --- | --- | --- | --- |
| **Is there one single professional you usually go to for most of your health problems?** | | | | Modified from CWF2016IHP |
|  | 1 | Yes, a doctor |  |  |
|  | 2 | Yes, another health care professional |  |  |
|  | 3 | No, there is no single health care professional I usually go for most of my health problems |  |  |
|  | 4 | Not sure |  |  |

ASK IF Q_CWF1130 = 1 NEW PAGE

Q_ USUALDR1 (SINGLE)

| **Questions** | | | | **Source** |
| --- | --- | --- | --- | --- |
| **Who is this doctor?** | | | | OECD PaRIS |
|  | 1 | A doctor specialised in family medicine/general practice |  |  |
|  | 2 | Another specialist doctor |  |  |
|  | 3 | Not sure |  |  |

**ASK IF Q_CWF1130 = 1 NEW PAGE** Q_CWF1140 (SINGLE)

| **Questions** | | | | **Source** |
| --- | --- | --- | --- | --- |
| **How long have you been seeing this doctor?** | | | | Modified from CWF2016IHP |
|  | 1 | 1 year or less |  |  |
|  | 2 | More than 1 year but no more than 3 years |  |  |
|  | 3 | More than 3 years but no more than 5 years |  |  |
|  | 4 | More than 5 years but no more than 10 years |  |  |
|  | 5 | More than 10 years |  |  |
|  | 6 | Not sure |  |  |

ASK IF Q_CWF1130 = 1 NEW PAGE

Q_ USUALDR2 (SINGLE)

| **Questions** | | | | **Source** |
| --- | --- | --- | --- | --- |
| **How many times have you seen this doctor in the past 12 months? This could be in person, on the phone, by video call or online messaging.** | | | | OECD PaRIS |
|  | 1 | Not in the past 12 months |  |  |
|  | 2 | 1 time |  |  |
|  | 3 | 2 times |  |  |
|  | 4 | 3 times |  |  |
|  | 5 | 4 times |  |  |
|  | 6 | 5 times or more |  |  |
|  | 7 | Not sure |  |  |

ASK IF Q_CWF1130 = 2 NEW PAGE

Q_ USUALDR3 (SINGLE)

| **Questions** | | | | **Source** |
| --- | --- | --- | --- | --- |
| **Who is this health care professional?** | | | | OECD PaRIS |
|  | 1 | A doctor specialised in family  medicine/general practice |  |  |
|  | 2 | Another specialist doctor |  |  |
|  | 3 | A nurse |  |  |
|  | 7 | Nurse practitioner |  |  |
|  | 4 | An allied health care professional, such as  a physiotherapist, dietician, or podiatrist |  |  |
|  | 5 | Another health care professional |  |  |
|  | 6 | Not sure |  |  |

ASK IF USUALDR3 = 1-3 NEW PAGE

Q_ USUALDR4 (SINGLE)

| **Questions** | | | | **Source** |
| --- | --- | --- | --- | --- |
| **How long have you been seeing this health care professional?** | | | | OECD PaRIS |
|  | 1 | 1 year or less |  |  |
|  | 2 | More than 1 year but no more than 3 years |  |  |
|  | 3 | More than 3 years but no more than 5 years |  |  |
|  | 4 | More than 5 years but no more than 10 years |  |  |
|  | 5 | More than 10 years |  |  |
|  | 6 | Not sure |  |  |

ASK IF USUALDR3 = 1-3 NEW PAGE

Q_ USUALDR5 (SINGLE)

| **Questions** | | | | **Source** |
| --- | --- | --- | --- | --- |
| **How many times have you seen this health care professional in the past 12 months? This could be in person, on the phone, by video call or online messaging.** | | | | OECD PaRIS |
|  | 1 | Not in the past 12 months |  |  |
|  | 2 | 1 time |  |  |
|  | 3 | 2 times |  |  |
|  | 4 | 3 times |  |  |
|  | 5 | 4 times |  |  |
|  | 6 | 5 times or more |  |  |
|  | 7 | Not sure |  |  |

SECTION 4 ‘Your experience of health care – part 1’ TIMING END

SECTION 5 ‘Your experience of health care – part 2’ TIMING START

ASK ALL NEW PAGE

Q_CHRONCOND (MULTI, OPEN)

| **Questions** | | | | **Source** |
| --- | --- | --- | --- | --- |
| **Have you ever been told by a doctor that you have any of the following health conditions?**  Please select all the options that apply. | | | | OECD PaRIS |
|  | 1 | High blood pressure |  |  |
|  | 2 | Cardiovascular or heart condition |  |  |
|  | 3 | Diabetes (type 1 or 2) |  |  |
|  | 4 | Arthritis or ongoing problem with back or joints |  |  |
|  | 5 | Breathing condition (e.g., asthma or COPD) |  |  |
|  | 6 | Alzheimer’s disease or other cause of dementia |  |  |
|  | 7 | Depression, anxiety or other mental health condition (e.g.,  bipolar disorder or schizophrenia) (ongoing) |  |  |
|  | 8 | Neurological condition (e.g., epilepsy or migraine) |  |  |
|  | 9 | Chronic kidney disease |  |  |
|  | 10 | Chronic liver disease |  |  |
|  | 11 | Cancer (diagnosis or treatment in the last 5 years) |  |  |
|  | 12 | Other long-term problem(s) |  |  |
|  | 13 | I have never been told by a doctor that I have any of these  problems |  |  |

ASK IF Q_CHRONCOND = 1 TO 12 NEW PAGE

The next few questions are about the care you may have received for these health conditions.

Please respond in relation to your experience of care and treatment by your << primary care provider>>.

Q_P3CEQ6 (SINGLE)

|  |  |
| --- | --- |
|  |  |
|  |  |
|  |  |

| **Questions** | **Source** |
| --- | --- |
| **Do you have a single professional who takes responsibility for coordinating your care across the services that you use?**   1. Yes 2. No 3. I do not receive care from more than one service 4. Not sure | Modified from Person-Centred Coordinated Care Experience  Questionnaire (P3CEQ) |

ASK IF Q_CHRONCOND = 1 TO 12 AND Q_P3CEQ6 = 1 NEW PAGE

Q_ P3CEQ5m1 (SINGLE)

| **Questions** | | | | **Source** |
| --- | --- | --- | --- | --- |
| **Is this the same professional that you see for most of your health problems?** | | | | OECD PaRIS |
|  | 1 | Yes |  |  |
|  | 2 | No |  |  |
|  | 3 | Not sure |  |  |

ASK IF Q_CHRONCOND = 1 TO 12 AND Q_P3CEQ5m1 = 2 OR 3 NEW PAGE

Q_P3CEQ5m2 (SINGLE)

| **Questions** | | | | **Source** |
| --- | --- | --- | --- | --- |
| **Who is this professional?** | | | | OECD PaRIS |
|  | 1 | A doctor specialised in family medicine/general practice |  |  |
|  | 2 | Another specialist doctor |  |  |
|  | 3 | A nurse |  |  |
|  | 7 | Nurse practitioner |  |  |
|  | 4 | An allied health care professional, such as a physiotherapist, dietician, or podiatrist |  |  |
|  | 5 | Another health care professional |  |  |
|  | 6 | Not sure |  |  |

ASK IF Q_CHRONCOND = 1 TO 12 NEW PAGE

Q_P3CEQ5 (SINGLE)

| **Questions** | | | | **Source** |
| --- | --- | --- | --- | --- |
| **Is your health care organised in a way that works for you?** | | | | Modified from Person-Centred Coordinated Care Experience Questionnaire (P3CEQ) |
|  | 1 | Yes, definitely |  |  |
|  | 2 | Yes, to some extent |  |  |
|  | 3 | No, not really |  |  |
|  | 4 | No, definitely not |  |  |
|  | 5 | Not sure |  |  |

ASK IF Q_CHRONCOND = 1 TO 12 NEW PAGE

Q_ROUTINEF (SINGLE)

| **Questions** | | | | **Source** |
| --- | --- | --- | --- | --- |
| **Are you offered regular follow-up for your health condition(s)? If you have more than one condition, please answer about the condition that you are seen for most regularly.** | | | | OECD PaRIS |
|  | 1 | Yes, about every 3 months or more often |  |  |
|  | 2 | Yes, about every 6 months |  |  |
|  | 3 | Yes, about every 12 months |  |  |
|  | 4 | Yes, over longer periods of time |  |  |
|  | 5 | No |  |  |
|  | 6 | Not sure |  |  |

ASK IF Q_CHRONCOND = 1 TO 12 NEW PAGE

Q_P3CE (LOOP)

|  |  |
| --- | --- |
|  |  |
|  |  |

| **Questions** | | | | **Source** |
| --- | --- | --- | --- | --- |
|  | | | | Modified from |
| P3CEQ1 **Do you discuss with the health care professionals** | | | | Person-Centred |
| **involved in your care what is most important for you in** | | | | Coordinated |
| **managing your own health and wellbeing?** | | | | Care Experience |
| P3CEQ2 **Are you involved as much as you want to be in** | | | | Questionnaire |
| **decisions about your care?** | | | | (P3CEQ) |
| P3CEQ3 **Are you considered as a ‘whole person’ rather than** | | | |  |
| **just a disease/condition in relation to your care?** | | | |  |
| Responses (SINGLE) | | | |  |
|  | 1 | Yes, definitely |  |  |
|  | 2 | Yes, to some extent |  |  |
|  | 3 | No, not really |  |  |
|  | 4 | No, definitely not |  |  |
|  | 5 | Not sure |  |  |

SECTION 5 ‘Your experience of health care – part 2’ TIMING END

SECTION 6 ‘Your experience of health care – part 3’ TIMING START

ASK IF Q_CHRONCOND = 1 TO 12 NEW PAGE

Sometimes health professionals support patients in defining health goals. These are things that are important to you and you want help in achieving.

Q_PCMH4 (SINGLE)

| **Questions** | | | | **Source** |
| --- | --- | --- | --- | --- |
| **How often does a health care professional talk to you about specific**  **goals for your health?** | | | | Modified from Patient-Centered Medical Home (PCMH) |
|  | 1 | Always |  |  |
|  | 2 | Often |  |  |
|  | 3 | Sometimes |  |  |
|  | 4 | Rarely |  |  |
|  | 5 | Never |  |  |
|  | 6 | Not sure |  |  |

ASK IF Q_CHRONCOND = 1 TO 12 NEW PAGE

The next few questions are about care plans. A care plan (or plan of care) is a written agreement between you and your health care professionals detailing the type of care you receive over an extended period of time and how this care will be given, also including what you will do yourself. Not everybody has a care plan.

Please respond in relation to your experience of care and treatment by your <<primary care provider>>.

ASK IF Q_CHRONCOND = 1 TO 12 NEW PAGE

Q_P3CEQ7a

|  |  |
| --- | --- |
|  |  |
|  |  |

| **Questions** | **Source** |
| --- | --- |
| **Do you have a care plan that takes into account all your health and wellbeing needs?**   1. Yes 2. No 3. Not sure | Person-Centred Coordinated Care Experience  Questionnaire (P3CEQ) |

ASK IF Q_CHRONCOND = 1 TO 12 AND Q_P3CEQ7a = 1 NEW PAGE

Q_P3CEQ7b (SINGLE)

|  |  |
| --- | --- |
|  |  |
|  |  |

| **Questions** | **Source** |
| --- | --- |
| **Is this care plan available to you?**   1. Yes 2. No 3. Not sure | Modified from Person-Centred Coordinated Care Experience Questionnaire  (P3CEQ) |

ASK IF Q_CHRONCOND = 1 TO 12 AND Q_P3CEQ7a = 1 NEW PAGE

P3CEQ7c (SINGLE)

| **Questions** | | | | **Source** |
| --- | --- | --- | --- | --- |
| **To what extent have you found your care plan useful for you to manage your health and wellbeing?** | | | | Modified from Person-Centred Coordinated Care Experience Questionnaire (P3CEQ) |
|  | 1 | Not at all |  |  |
|  | 2 | To some extent |  |  |
|  | 3 | To a large extent |  |  |
|  | 4 | Completely |  |  |
|  | 5 | Not sure |  |  |

ASK IF Q_CHRONCOND = 1 TO 12 AND Q_P3CEQ7a = 1 NEW PAGE

P3CEQ7d (SINGLE)

| **Questions** | | | | **Source** |
| --- | --- | --- | --- | --- |
| **To what extent do all the professionals involved in your care appear to be following the same care plan?** | | | | Modified from Person-Centred Coordinated Care Experience Questionnaire (P3CEQ) |
|  | 1 | Not at all |  |  |
|  | 2 | To some extent |  |  |
|  | 3 | To a large extent |  |  |
|  | 4 | Completely |  |  |
|  | 5 | Not sure |  |  |

ASK IF Q_CHRONCOND = 1 TO 12 NEW PAGE

Q_P3CEQ8 (SINGLE)

| **Questions** | | | | **Source** |
| --- | --- | --- | --- | --- |
| **Do you have enough support from your health care professionals to help you to manage your own health and wellbeing?** | | | | Modified from Person-Centred Coordinated Care Experience Questionnaire (P3CEQ) |
|  | 1 | I do not need support |  |  |
|  | 2 | I have had no support |  |  |
|  | 3 | I sometimes have enough support |  |  |
|  | 4 | I often have enough support |  |  |
|  | 5 | I always have enough support |  |  |
|  | 6 | Not relevant |  |  |
|  | 7 | Not sure |  |  |

ASK IF Q_CHRONCOND = 1 TO 12 NEW PAGE

P3CEQ9 (SINGLE)

| **Questions** | | | | **Source** |
| --- | --- | --- | --- | --- |
| **To what extent do you receive useful information at the time you need it to help you manage your health and wellbeing?** | | | | Modified from Person-Centred Coordinated Care Experience Questionnaire (P3CEQ) |
|  | 1 | I do not receive any information |  |  |
|  | 2 | I sometimes receive enough information |  |  |
|  | 3 | I often receive enough information |  |  |
|  | 4 | I always receive enough information |  |  |
|  | 5 | I receive too much information |  |  |
|  | 6 | Not relevant |  |  |
|  | 7 | Not sure |  |  |

ASK IF Q_CHRONCOND = 1 TO 12 NEW PAGE

Q_P3CEQ11b (SINGLE)

| **Questions** | | | | **Source** |
| --- | --- | --- | --- | --- |
| **Do your health care professionals involve your family, friends and/or carers as much as you want them to be in decisions about your care?** | | | | Modified from Person-Centred Coordinated Care Experience Questionnaire (P3CEQ) |
|  | 1 | No, definitely not |  |  |
|  | 2 | No, not really |  |  |
|  | 3 | Yes, to some extent |  |  |
|  | 4 | Yes, definitely |  |  |
|  | 5 | I do not want my family, friends or carers to be involved |  |  |
|  | 6 | My family, friends or carers do not want to be involved |  |  |
|  | 7 | Not applicable |  |  |

ASK IF Q_CHRONCOND = 1 TO 12 NEW PAGE

Q_MEDCWF1605 (SINGLE)

| **Questions** | | | | **Source** |
| --- | --- | --- | --- | --- |
| **How many different medications as prescribed by a doctor or a nurse are you taking on a regular or ongoing basis?** | | | | Modified from CWF2016IHP |
|  | 1 | No medication |  |  |
|  | 2 | 1-2 medications |  |  |
|  | 3 | 3-4 medications |  |  |
|  | 4 | 5-9 medications |  |  |
|  | 5 | 10 or more medications |  |  |

ASK IF Q_CHRONCOND = 1 TO 12 AND Q_MEDCWF1605 = 2 TO 5 NEW PAGE

Q_MEDCWF71110 (SINGLE)

| **Questions** | | | | **Source** |
| --- | --- | --- | --- | --- |
| **In the past 12 months, has a health care professional reviewed with you all medications you take?** | | | | Modified from CWF2017IHP |
|  | 1 | Yes |  |  |
|  | 2 | No |  |  |
|  | 3 | Not sure |  |  |

SECTION 6 ‘Your experience of health care – part 3’ TIMING END

SECTION 7 ‘Your experience of health care – part 4’ TIMING START

ASK ALL NEW PAGE

The next few questions are about your experience with your <<primary care provider>> in the last 12 months.

Q_ P3CEQ4 (SINGLE)

| **Questions** | | | | **Source** |
| --- | --- | --- | --- | --- |
| **Were there times when you had to repeat information that should have been in your care records?** | | | | Modified from Person-Centred Coordinated Care Experience Questionnaire (P3CEQ) |
|  | 1 | Yes, definitely |  |  |
|  | 2 | Yes, to some extent |  |  |
|  | 3 | No, not really |  |  |
|  | 4 | No, definitely not |  |  |
|  | 5 | Not sure |  |  |

ASK ALL NEW PAGE

Q_PREOS3 (SINGLE)

Sometimes patients experience an event or circumstance that could have resulted, or did result, in unnecessary harm to themselves, such as not getting an appointment when needed; receiving a wrong or delayed diagnosis or treatment; or experiencing problems with communications between health care professionals.

| **Questions** | | | | **Source** |
| --- | --- | --- | --- | --- |
| **How often do you believe you have had any such event or circumstance with your primary care centre?** | | | | Modified from PREOS-PC-6 |
|  | 1 | Always |  |  |
|  | 2 | Often |  |  |
|  | 3 | Sometimes |  |  |
|  | 4 | Rarely |  |  |
|  | 5 | Never |  |  |
|  | 98 | Not applicable |  |  |

ASK ALL NEW PAGE

Q_PREOS1 (SINGLE)

| **Questions** | | | | **Source** |
| --- | --- | --- | --- | --- |
| **How often did you feel that the health care professionals at your primary care centre encouraged you to talk about any concerns about your health care?** | | | | Modified from PREOS-PC-6 |
|  | 1 | Always |  |  |
|  | 2 | Often |  |  |
|  | 3 | Sometimes |  |  |
|  | 4 | Rarely |  |  |
|  | 5 | Never |  |  |
|  | 98 | Not applicable |  |  |

ASK ALL NEW PAGE

Q_OECDACC3 (SINGLE)

| **Questions** | | | | **Source** |
| --- | --- | --- | --- | --- |
| **How often did you have a health problem but did not seek care because of difficulties in travelling to your primary care centre?** | | | | Modified from OECD  proposed Set of Questions on Patient Experiences with Ambulatory Care |
|  | 1 | Always |  |  |
|  | 2 | Often |  |  |
|  | 3 | Sometimes |  |  |
|  | 4 | Rarely |  |  |
|  | 5 | Never |  |  |
|  | 98 | Not applicable |  |  |

ASK ALL NEW PAGE

Q_ OECDACC46 (SINGLE)

| **Questions** | | | | **Source** |
| --- | --- | --- | --- | --- |
| **How often did you have a health problem but did not seek care, or did not take a prescription medicine because of the cost?** | | | | Modified from OECD  proposed Set of Questions on Patient Experiences with Ambulatory Care |
|  | 1 | Always |  |  |
|  | 2 | Often |  |  |
|  | 3 | Sometimes |  |  |
|  | 4 | Rarely |  |  |
|  | 5 | Never |  |  |
|  | 98 | Not applicable |  |  |

ASK ALL NEW PAGE

The next questions are about the online services that your primary care centre may offer to patients like you, such as a website, smartphone app or video consultation

ASK ALL NEW PAGE

Q_GPPS4 (SINGLE)

| **Questions** | | | | **Source** |
| --- | --- | --- | --- | --- |
| **How easy is it for you to use your primary care centre’s website to look for information or access services?** | | | | GP Patient Survey (GPPS) |
|  | 1 | Very easy |  |  |
|  | 2 | Fairly easy |  |  |
|  | 3 | Not very easy |  |  |
|  | 4 | Not at all easy |  |  |
|  | 5 | I haven’t tried |  |  |
|  | 6 | My primary care centre does not have a website |  |  |
|  | 7 | Not sure |  |  |

ASK ALL NEW PAGE

Q_GPPS5 (MULTI)

| **Questions** | | | | **Source** |
| --- | --- | --- | --- | --- |
| **As far as you know, which of the following online services does your primary care centre offer?**  Please select all the options that apply. | | | | Modified from GP Patient Survey (GPPS) |
|  | 1 | Booking appointments online |  |  |
|  | 2 | Ordering repeat prescriptions online |  |  |
|  | 3 | Accessing my medical records online |  |  |
|  | 4 | Video consultations |  |  |
|  | 5 | None of these |  |  |
|  | 99 | Don’t know |  |  |

ASK ALL NEW PAGE

Q_GPPS6 (MULTIPLE)

| **Questions** | | | | **Source** |
| --- | --- | --- | --- | --- |
| **Which of the following online services offered by your primary care centre have you used?**  Please select all the options that apply. | | | | Modified from GP Patient Survey (GPPS) |
|  | 1 | Booking appointments online |  |  |
|  | 2 | Ordering repeat prescriptions online |  |  |
|  | 3 | Accessing my medical records online |  |  |
|  | 4 | Video consultations |  |  |
|  | 5 | None of these |  |  |

ASK ALL NEW PAGE

Q_CWF119 (SINGLE)

| **Questions** | | | | **Source** |
| --- | --- | --- | --- | --- |
| **Overall, how do you rate the medical care that you have received in the past 12 months from your primary care centre?** | | | | Modified from CWF2016IHP |
|  | 1 | Excellent |  |  |
|  | 2 | Very good |  |  |
|  | 3 | Good |  |  |
|  | 4 | Fair |  |  |
|  | 5 | Poor |  |  |
|  | 6 | Have not received medical care in the last 12 months |  |  |
|  | 7 | Not sure |  |  |

SECTION 7 ‘Your experience of health care – part 4’ TIMING END

SECTION 8 ‘Other health services’ TIMING START

ASK ALL NEW PAGE

#### Other health services

The next few questions are about other health services you have used in the last 12 months.

ASK ALL NEW PAGE

Q_ EMERG (SINGLE)

| **Questions** | | | | **Source** |
| --- | --- | --- | --- | --- |
| **In the last 12 months, have you been to a hospital emergency department for your own medical care?** | | | | Modified from CWF2017IHP |
|  | 1 | No |  |  |
|  | 2 | Yes, once |  |  |
|  | 3 | Yes, twice |  |  |
|  | 4 | Yes, 3 or more times |  |  |
|  | 5 | Not sure |  |  |

ASK ALL NEW PAGE

Q_CWF2016HOSP (SINGLE)

| **Questions** | | | | **Source** |
| --- | --- | --- | --- | --- |
| **In the last 12 months, have you been in a hospital for one night or**  **longer?** | | | | Modified from CWF2016IHP |
|  | 1 | No |  |  |
|  | 2 | Yes, once |  |  |
|  | 3 | Yes, twice |  |  |
|  | 4 | Yes, 3 or more times |  |  |
|  | 5 | Not sure |  |  |

SECTION 8 ‘Other health services’ TIMING END

SECTION 9 ‘Your last consultation’ TIMING START

ASK ALL NEW PAGE

Your last consultation **(SHOW ON SCREEN FOR ALL QS UP TO Q_OECDEXP5)**

The next questions are about the last time that you had a consultation with your <<primary care provider>> with a doctor, nurse or other health care professional to get care for yourself. This may have been with your main health care professional, or another health care professional, and includes consultations over the phone or in a doctor’s office or clinic.

ASK ALL NEW PAGE

LAST APP (country specific question to establish eligibility)

ASK ALL NEW PAGE

Q_PURPOS (SINGLE)

| **Questions** | | | | **Source** |
| --- | --- | --- | --- | --- |
| **What was the main purpose of this consultation?** | | | | OECD PaRIS |
|  | 1 | First contact for a new problem |  |  |
|  | 2 | Follow-up of a new problem |  |  |
|  | 3 | Routine care for a long-term problem |  |  |
|  | 4 | Non-routine care for a long-term problem |  |  |
|  | 5 | General preventive care |  |  |
|  | 6 | Not sure |  |  |

ASK ALL NEW PAGE

Q_OECDACC7a (SINGLE)

| **Questions** | | | | **Source** |
| --- | --- | --- | --- | --- |
| **Who did you have this last consultation with? If you saw more than one health care professional, please select the one you spent most time with on the day. Please select only one option.** | | | | Modified from OECD  proposed Set of Questions on Patient Experiences with Ambulatory Care |
|  | 1 | A doctor specialised in family  medicine/general practice |  |  |
|  | 2 | Another specialist doctor |  |  |
|  | 3 | A nurse |  |  |
|  | 7 | Nurse practitioner |  |  |
|  | 4 | An allied health care professional, such as  a physiotherapist, dietician, or podiatrist |  |  |
|  | 5 | Another health care professional |  |  |
|  | 6 | Not sure |  |  |

ASK ALL NEW PAGE

Q_OECDACC7b (SINGLE)

|  |  |
| --- | --- |
|  |  |
|  |  |
|  |  |
|  |  |

| **Questions** | **Source** |
| --- | --- |
| **Where was this health care professional working?** | Modified from |
| 1 A primary care centre | OECD |
| 2 An outpatient department of a hospital | proposed Set |
| 1. Other 2. Not sure 3. Not applicable | of Questions on Patient Experiences  with |
|  | Ambulatory |
|  | Care |

ASK ALL NEW PAGE

Q_OECDACC7c (MULTI)

| **Questions** | | | | **Source** |
| --- | --- | --- | --- | --- |
| **Which of the following best describes the type of care you received?**  Please select all the options that apply. | | | | Modified from OECD  proposed Set of Questions on Patient Experiences with Ambulatory Care |
|  | 1 | Face-to-face consultation in the health care  professional’s office |  |  |
|  | 2 | Telephone consultation |  |  |
|  | 3 | Video consultation |  |  |
|  | 4 | Home visit |  |  |
|  | 5 | Other |  |  |
|  | 6 | Not sure |  |  |

ASK ALL NEW PAGE

GPPSACCESS (SINGLE)

| **Questions** | | | | **Source** |
| --- | --- | --- | --- | --- |
| **How long after initially trying to book the appointment did the**  **appointment take place?** | | | | Modified from GP Patient Survey (GPPS) |
|  | 1 | On the same day |  |  |
|  | 2 | On the next day |  |  |
|  | 3 | A few days and up to a week later |  |  |
|  | 4 | More than a week and up to one month  later |  |  |
|  | 5 | More than one month later |  |  |
|  | 6 | Can’t remember |  |  |

ASK ALL NEW PAGE

Q_OECDACC9 (SINGLE)

|  |  |
| --- | --- |
|  |  |
|  |  |

| **Questions** | **Source** |
| --- | --- |
| **Was the time you waited a problem for you?**   1. Yes 2. No 3. Not sure | Modified from OECD  proposed Set of Questions on Patient Experiences with Ambulatory  Care |

ASK ALL NEW PAGE

Q_OECDACC8 (SINGLE)

| **Questions** | | | | **Source** |
| --- | --- | --- | --- | --- |
| **Did this health care professional spend enough time with you?** | | | | OECD  proposed Set of Questions on Patient Experiences with Ambulatory Care |
|  | 1 | Yes, definitely |  |  |
|  | 2 | Yes, to some extent |  |  |
|  | 3 | No, not really |  |  |
|  | 4 | No, definitely not |  |  |
|  | 5 | Not sure |  |  |

ASK ALL NEW PAGE

Q_OECDEXP2 (SINGLE)

| **Questions** | | | | **Source** |
| --- | --- | --- | --- | --- |
| **Did this health care professional explain things in a way that was easy to understand?** | | | | OECD  proposed Set of Questions on Patient Experiences with Ambulatory Care |
|  | 1 | Yes, definitely |  |  |
|  | 2 | Yes, to some extent |  |  |
|  | 3 | No, not really |  |  |
|  | 4 | No, definitely not |  |  |
|  | 5 | Not sure |  |  |

ASK ALL NEW PAGE

Q_GPPSTRUST (SINGLE)

| **Questions** | | | | **Source** |
| --- | --- | --- | --- | --- |
| **Did you have confidence and trust in the health care professional you saw or spoke to?** | | | | Modified from GP Patient Survey (GPPS) |
|  | 1 | Yes, definitely |  |  |
|  | 2 | Yes, to some extent |  |  |
|  | 3 | No, not really |  |  |
|  | 4 | No, definitely not |  |  |
|  | 5 | Not sure |  |  |

ASK ALL NEW PAGE

Q_OECDEXP5 (SINGLE)

| **Questions** | | | | **Source** |
| --- | --- | --- | --- | --- |
| **Overall, how would you rate the quality of this consultation?** | | | | OECD  proposed Set of Questions on Patient Experiences with Ambulatory Care |
|  | 1 | Excellent |  |  |
|  | 2 | Very good |  |  |
|  | 3 | Good |  |  |
|  | 4 | Fair |  |  |
|  | 5 | Poor |  |  |
|  | 6 | Not sure |  |  |

SECTION 9 ‘Your last consultation’ TIMING END

SECTION 10 ‘COVID-19’ TIMING START

ASK ALL NEW PAGE

The next questions are about if you have tested positive for COVID-19 and symptoms you may have experienced.

Q_COVID1 (SINGLE)

| **Questions** | | | | **Source** |
| --- | --- | --- | --- | --- |
| **Have you ever tested positive for COVID-19 (using a rapid point-of-care test, self-test, or laboratory test) or been told by a doctor or other health care provider that you have or had COVID-19?** | | | | Modified from Household Pulse Survey US |
|  | 1 | Yes |  |  |
|  | 2 | No |  |  |
|  | 3 | Not sure |  |  |

ASK IF Q_COVID1 = 1

Q_COVID2 (SINGLE)

| **Questions** | | | | **Source** |
| --- | --- | --- | --- | --- |
| **Did you have any symptoms lasting 2 months or longer that you did not have prior to having coronavirus or COVID-19?**  *Long term symptoms may include: tiredness or fatigue, difficulty thinking, concentrating, forgetfulness, or memory problems (sometimes referred to as "brain fog", difficulty breathing or shortness of breath, joint or muscle pain, fast-beating or pounding heart (also known as heart palpitations), chest pain, dizziness on standing, menstrual changes, changes to taste/smell, or inability to exercise.* | | | | Modified from Household Pulse Survey US |
|  | 1 | Yes, symptoms lasted between 2-3 months |  |  |
|  | 2 | Yes, symptoms lasted between 3-6 months |  |  |
|  | 3 | Yes, symptoms lasted between 6 months - 1 year |  |  |
|  | 4 | Yes, symptoms lasted at least 1 year |  |  |
|  | 5 | No |  |  |
|  | 6 | Not sure |  |  |

ASK IF Q_COVID2 = 1 TO 4

Q_COVID3 (SINGLE)

| **Questions** | | | | **Source** |
| --- | --- | --- | --- | --- |
| **Do you still have these long-term symptoms?** | | | | Modified from Household Pulse Survey US |
|  | 1 | Yes |  |  |
|  | 2 | No |  |  |
|  | 3 | Not sure |  |  |

ASK IF Q_COVID3 = 1

Q_COVID4 (SINGLE)

| **Questions** | | | | **Source** |
| --- | --- | --- | --- | --- |
| **Do these long-term symptoms reduce your ability to carry out day-to-day activities compared with the time before you had COVID-19?** | | | | Modified from Household Pulse Survey US |
|  | 1 | Yes, a lot |  |  |
|  | 2 | Yes, a little |  |  |
|  | 3 | Not at all |  |  |
|  | 3 | Not sure |  |  |

SECTION 10 ‘COVID-19’ TIMING END

SECTION 11 ‘About yourself’ TIMING START

ASK ALL NEW PAGE

About yourself

Finally, we would like to ask you some information about yourself. It is important to collect this information to understand whether there are any differences in the quality of services experienced by different groups of people.

**ASK ALL NEW PAGE** Q_WB151

| **Questions** | | | | **Source** |
| --- | --- | --- | --- | --- |
| **How much do you weigh?** | | | | Modified from WELL-BEING QUESTIONNAIRE FOR PISA 2018  (INTERNATIONAL OPTION) |
|  | 1 | kilos (MIN 20, MAX 500) (ALTERNATIVE IN IMPERIAL) |  |  |
|  | 99 | Don’t know |  |  |
|  | 97 | Prefer not to say |  |  |

ASK ALL NEW PAGE

Q_WB152 (SINGLE)

| **Questions** | | **Source** |
| --- | --- | --- |
| **How tall are you?** | | Modified from WELL-BEING QUESTIONNAIRE FOR PISA  2018(INTERNATIO NAL OPTION) |
| 1 | centimeters (MIN 50, MAX 250) (ALTERNATIVE IN IMPERIAL) |  |
| 99 | Don’t know |  |
| 97 | Prefer not to say |  |

ASK ALL NEW PAGE

Q_ISCED (SINGLE)

|  |  |
| --- | --- |
|  |  |
|  |  |
|  |  |
|  |  |
|  |  |
|  |  |
|  |  |
|  |  |

| **Questions** | **Source** |
| --- | --- |
| **What is the highest educational level that you have attained?**   1. Early childhood education 2. Primary education 3. Lower secondary education 4. Upper secondary education 5. Post-secondary non-tertiary education 6. Short-cycle tertiary education 7. Bachelor’s degree or equivalent level 8. Master’s degree or equivalent level 9. Doctoral or equivalent level | Modified from International Standard Classification of Education (ISCED) |

ASK ALL NEW PAGE

Q_SEX (SINGLE, OPEN)

| **Questions** | | | | **Source** |
| --- | --- | --- | --- | --- |
| **Which of the following best describes you?** | | | | Modified from OECD Risks That Matter |
|  | 1 | Female |  |  |
|  | 2 | Male |  |  |
|  | 3 | LEAVE BLANK |  |  |
|  | 4 | Other |  |  |
|  | 97 | Prefer not to say |  |  |

ASK ALL NEW PAGE

Q_GENDERID (COUNTRY SPECIFIC QUESTION)

ASK ALL NEW PAGE

Q_LGBT (SINGLE)

| **Questions** | | | | **Source** |
| --- | --- | --- | --- | --- |
| **This question is about your sexual orientation. Do you identify as:** | | | | Modified from UK National LGBT Survey |
|  | 1 | Heterosexual or straight (that is, attracted to the opposite  sex) |  |  |
|  | 2 | Homosexual (gay or lesbian, that is, attracted to the same  sex) |  |  |
|  | 3 | Bisexual (attracted to both sexes) |  |  |
|  | 4 | Other |  |  |
|  | 97 | Prefer not to say |  |  |

ASK ALL NEW PAGE

Q_OECDLIT5a (SINGLE)

| **Questions** | | | | **Source** |
| --- | --- | --- | --- | --- |
| **Which of these terms best describes your current work situation?** | | | | OECD  Measuring Financial Literacy |
|  | 1 | Self-employed [work for yourself] |  |  |
|  | 2 | In paid employment [work for someone else] |  |  |
|  | 3 | Looking for work |  |  |
|  | 4 | Looking after the home |  |  |
|  | 5 | Unable to work due to sickness or ill-health |  |  |
|  | 6 | Retired |  |  |
|  | 7 | Student |  |  |
|  | 8 | Not working and not looking for work |  |  |
|  | 9 | Apprentice |  |  |
|  | 10 | Other |  |  |
|  | 99 | Don’t know |  |  |

ASK ALL NEW PAGE

Q_OECDLIT7 (SINGLE)

| **Questions** | | | | **Source** |
| --- | --- | --- | --- | --- |
| **Which of these categories does your household net income usually fall into?** | | | | Modified from OECD  Measuring Financial Literacy |
|  | 1 | Up to $X a month |  |  |
|  | 2 | Between $X and $Y a month |  |  |
|  | 3 | $Y or more a month |  |  |
|  | 99 | Don’t know |  |  |
|  | 97 | Prefer not to say |  |  |

ASK ALL NEW PAGE

Q_MONMED (SINGLE)

| **Questions** | | | | **Source** |
| --- | --- | --- | --- | --- |
| **In the past 12 months, did you have problems paying or were unable to pay any medical bills?** | | | | National Health Interview Survey (NHIS) |
|  | 1 | Yes |  |  |
|  | 2 | No |  |  |
|  | 98 | Not applicable |  |  |

ASK ALL NEW PAGE

Q_MON (Show on different screens)

|  |  |
| --- | --- |
|  |  |
|  |  |

| **Questions** | | | | **Source** |
| --- | --- | --- | --- | --- |
| **How often in the past 12 months would you say you were worried or stressed about the following things?**  MONMEAL Having enough money to buy healthy meals? MONRENT Having enough money to pay your rent or mortgage? MONBILLS Having enough money to pay for other monthly bills,  like electricity, heat, and your telephone?  Responses (SINGLE) | | | | Modified from 2017  Commonwealth Fund International Health Policy Survey of Older Adults |
|  | 1 | Always |  |  |
|  | 2 | Often |  |  |
|  | 3 | Sometimes |  |  |
|  | 4 | Rarely |  |  |
|  | 5 | Never |  |  |

**ASK ALL NEW PAGE** MEDHIMS6

|  |  |
| --- | --- |
|  |  |

| **Questions** | **Source** |
| --- | --- |
| **Were you born in (SURVEY COUNTRY)?** (SINGLE, OPEN)   1. Yes 2. No (Please state the country you were born in   ) | Mediterranean Household International Migration Survey (MED-  HIMS) |

**ASK ALL NEW PAGE** MEDHIMS7

|  |  |
| --- | --- |
|  |  |

| **Questions** | **Source** |
| --- | --- |
| **Are you a citizen of (SURVEY COUNTRY)?** (SINGLE, OPEN)   1. Yes 2. No (Please state what country you are a citizen of   ) | Mediterranean Household International Migration Survey (MED-HIMS) |

**ASK ALL NEW PAGE** Q_OECDLITii

| **Questions** | | | | **Source** |
| --- | --- | --- | --- | --- |
| **Which of these best describes the type of area in which you live?** | | | | Modified from OECD  Measuring Financial Literacy |
|  | 1 | City |  |  |
|  | 2 | Town or suburb |  |  |
|  | 3 | Rural area |  |  |
|  | 4 | Don’t know |  |  |

ASK ALL NEW PAGE

OECDLIT2a (OPEN)

| **Questions** | **Source** |
| --- | --- |
| **How many children under the age of 18 live with you, in your household?**  WRITE IN NUMBER (MIN 0, MAX 20) | OECD  Measuring Financial  Literacy |

ASK ALL NEW PAGE

OECDLIT2b (OPEN)

| **Questions** | **Source** |
| --- | --- |
| **How many people aged 18 and over live with you, in your household? Please do not count yourself**  WRITE IN NUMBER (MIN 0, MAX 20) | OECD  Measuring  Financial Literacy |

ASK ALL NEW PAGE

WHODIS (Show on different screens)

|  |  |
| --- | --- |
|  |  |

| **Questions** | | | | **Source** |
| --- | --- | --- | --- | --- |
| **Should you need help, how easy is it for you to get help from the** | | | | Modified from |
| **following people?** | | | | WHO/ World |
| WHODIS1 A close family member (including your partner)? WHODIS2 Friends, neighbours and co-workers? | | | | Bank model-disability-survey |
| Responses (SINGLE) | | | |  |
|  | 1 | Very easy |  |  |
|  | 2 | Easy |  |  |
|  | 3 | Neither easy nor difficult |  |  |
|  | 4 | Difficult |  |  |
|  | 5 | Very difficult |  |  |
|  | 6 | Not applicable |  |  |

ASK ALL NEW PAGE

WHOWB11 (SINGLE)

| **Questions** | | | | **Source** |
| --- | --- | --- | --- | --- |
| **Do you need physical care or support, such as help with eating, dressing, bathing, moving around the house or assistance outside the house such as for using transportation?** | | | | Modified from WHO/ World Bank model-disability-survey |
|  | 1 | Yes |  |  |
|  | 2 | No |  |  |

ASK ALL NEW PAGE

Q_WHOWB12 (SINGLE)

| **Questions** | | | | **Source** |
| --- | --- | --- | --- | --- |
| **Do you need emotional care or support, such as comfort, advice or counseling?** | | | | Modified from WHO/ World Bank model-disability-survey |
|  | 1 | Yes |  |  |
|  | 2 | No |  |  |

ASK ALL NEW PAGE

Q_WHOWB13 (SINGLE)

| **Questions** | | | | **Source** |
| --- | --- | --- | --- | --- |
| **Do you need support for health care, such as administering medicines, changing bandages or arranging for appointments with primary care providers?** | | | | Modified from WHO/ World Bank model-disability-survey |
|  | 1 | Yes |  |  |
|  | 2 | No |  |  |

**ASK ALL NEW PAGE** Q_TRUST

| **Questions** | | | | **Source** |
| --- | --- | --- | --- | --- |
| **How strongly do you agree or disagree that the health care system can be trusted?** | | | | OECD PaRIS |
|  | 1 | Strongly disagree |  |  |
|  | 2 | Disagree |  |  |
|  | 3 | Neither agree nor disagree |  |  |
|  | 4 | Agree |  |  |
|  | 5 | Strongly agree |  |  |

ASK ALL NEW PAGE

Q_RESPONDENT

| **Questions** | | | | **Source** |
| --- | --- | --- | --- | --- |
| **Who was the main person or people that filled in this questionnaire?** | | | | NHS Inpatient Survey 2022 |
|  | 1 | The person invited to complete the survey |  |  |
|  | 2 | A friend or relative of the person invited to complete the survey |  |  |
|  | 3 | Both the person invited to complete the survey and a friend or relative together |  |  |
|  | 4 | The person invited to complete the survey with the help of a health care professional or care worker |  |  |

# Thank you for taking the time to complete this questionnaire

SECTION 11 ‘About yourself’ TIMING END

## Annex A. Source questionnaires of the PaRIS-PQ

| **Source** | **Reference** |
| --- | --- |
| Commonwealth Fund (CWF) Surveys: 2016 IHP, 2017 IHP | The Commonwealth Fund (2016), *International Health Policy Survey*. The Commonwealth Fund (2017), *International Health Policy Survey*. |
| eHealth Literacy Scale (eHEALS) | Norman CD, Skinner HA (2006a), eHEALS: The eHealth literacy scale. *Journal of Medical Internet Research*, *8*(4). |
| European Health Interview 2020 Edition | Eurostat and European Commission (2020), *European Health Interview Survey (EHIS wave 3) Methodological*  *manual*. Luxembourg: Publications Office of the European Union. |
| GP Patient Survey (GPPS) | Campbell J, Smith P, Nissen S, Bower P, Elliott M, Roland M. The GP Patient Survey for use in primary care in the National Health Service in the UK—development and psychometric characteristics. *BMC Fam Pract*. 2009;10:57.  <https://www.gp-patient.co.uk/> |
| Household Pulse Survey US | United States Census Bureau (2023), *Phase 3.7 Household Pulse Survey*.  [https://www.census.gov/programs-surveys/household-pulse-survey/technical-documentation/questionnaires.2023.html#phase3.](https://www.census.gov/programs-surveys/household-pulse-survey/technical-documentation/questionnaires.2023.html#phase3)7 |
| International Standard Classification of Education (ISCED) | UNESCO Institute for Statistics (2011), *International Standard Classification of Education ISCED 2011* |
| International Physical Activity Questionnaire - Short Form (IPAQ-SF) | Craig CL, Marshall AL, Sjostrom M, Bauman A, Booth ML, Ainsworth BE, Pratt M, Ekelund U, Yngve A, Sallis JF, Oja P: International Physical Activity Questionnaire: 12-country reliability and validity. *Medicine and Science in Sports and Exercise*. 2003, 35: 1381-1395. |
| Medicare Patient Engagement Questions | Parker JL, Regan JF, Petroski J (2014), Beneficiary activation in the Medicare population. *Medicare & Medicaid Research Review*, 4, E1-E14.  <https://www.cms.gov/data-research/research/medicare-current-beneficiary-survey> |
| Mediterranean Household  International Migration Survey (MED-HIMS) | The European Union (2019), *Mediterranean Household International Migration Survey (MED-HIMS)*. |
| National Health Interview Survey (NHIS) | Cohen RA, Cha AE (2023), *Problems paying medical bills: United States, 2021.* |
| NHS Inpatient Survey 2022 | National Health Service, Care Quality Commission (2022), *NHS inpatient survey* |
| OECD Measuring Financial Literacy | OECD INFE (2011) Measuring Financial Literacy: Core Questionnaire in  *Measuring Financial Literacy: Questionnaire and Guidance Notes for conducting an Internationally Comparable Survey of Financial literacy.* |

|  | Paris: OECD. |
| --- | --- |
| OECD proposed Set of Questions on Patient Experiences with Ambulatory Care | OECD (2018), *OECD Health Working Papers No. 102*. |
| OECD Risks That Matter | OECD (2018), *OECD Risks That Matter Core Questionnaire*. |
| Patient-Centered Medical Home (PCMH) | Measures From the CAHPS Patient-Centered Medical Home Item Set. Content last reviewed May 2017. Agency for Healthcare Research and Quality, Rockville, MD.  <https://www.ahrq.gov/cahps/surveys-guidance/item-sets/pcmh/measures.html> |
| Person-Centred Coordinated Care Experience Questionnaire (P3CEQ)* | Lloyd H, Fosh B, Whalley B, Byng R, Close J (2019), Validation of the person-centred coordinated care experience questionnaire (P3CEQ). *International Journal for Quality in Health Care*, *31*(7), 506-512.  *The P3CEQ is copyright [2018] University of Plymouth. All rights reserved. |
| PISA-D Main Study Teacher Questionnaire IS | OECD (2016) *OECD PISA-D Main Study Teacher Questionnaire IS* |
| Porter-Novelli Consumer Preferences Scale | Maibach EW, Weber D, Massett H, Hancock GR, Price S (2006), Understanding consumers' health information preferences development and validation of a brief screening instrument. *Journal of health communication*, *11*(8), 717-736. |
| Patient Reported Experiences and Outcomes of Safety in Primary Care (PREOS-PC-6) | Mounce L, Gangannagaripalli J, Ricci-Cabello I, Avery AJ, Valderas JM (2017, October). Development of two short versions of the Patient Reported Experiences and Outcomes of Safety in Primary Care (PREOS-PC) questionnaire: PREOS-PC compact and PREOS-PC-6.  Q*uality of Life Research* (Vol. 26, No. 1, pp. 110-110). |
| PROMIS® Scale v1.2 – Global Health | Available from <https://www.promishealth.org/57461-2/> |
| PROMIS Adult Short Form v1.0 - Pain Interference | Available from <https://www.promishealth.org/57461-2/> |
| PROMIS Item Bank v. 1.0 – Dyspnea | Available from <https://www.promishealth.org/57461-2/> |
| UK National LGBT Survey | Department of Education (2018), *National LGBT Research Report*.  Available from <https://www.gov.uk/government/publications/national-lgbt-survey-summary-report> |
| Well-being questionnaire for PISA 2018 | OECD (2018), *Well-being questionnaire for Pisa*. |
| WHO/ World Bank model-disability-survey | World Health Organization (2017) *Model Disability Survey*.  Available from <https://www.who.int/publications/i/item/> 9789241512862. |
| WHO Well-being Index (WHO-5) | Heun R, Bonsignore M, Barkow K, Jessen F (2001), Validity of the five-item WHO Well-Being Index (WHO-5) in an elderly population. *European archives of psychiatry and clinical neuroscience*, *251*, 27-31. |

**Supplementary File B**

**GRIPP2 Checklist (short form)**

| **Section and topic** | **Item** | **Reported on page No** |
| --- | --- | --- |
| **1: Aim** | Report the aim of PPI in the study | Under Rationale/ specific objectives starting from page 8. |
| **2 : Methods** | Provide a clear description of the methods used for PPI in the study | Reported under Methods on page 9. |
| **3: Study results** | Outcomes—Report the results of PPI in the study, including both positive and negative outcomes | Reported under Findings on page 15. |
| **4: Discussion and conclusions** | Outcomes—Comment on the extent to which PPI influenced the study overall. Describe positive and negative effects | Reported under Discussions and Conclusions starting from page 21 |
| **5: Reflections/critical perspective** | Comment critically on the study, reflecting on the things that went well and those that did not, so others can learn from this experience | Reported under Discussions (from the second paragraph) on page 22. |

**Supplementary File C**

# **Revised Standards for Quality Improvement Reporting Excellence (SQUIRE 2.0) September 15, 2015**

| **Text Section and Item**  **Name** | **Section or Item Description** |  |
| --- | --- | --- |
| **Notes to authors** | - The SQUIRE guidelines provide a framework for reporting new   knowledge about how to improve healthcare   - The SQUIRE guidelines are intended for reports that describe [system](#_bookmark13) level work to improve the quality, safety, and value of healthcare, and used methods to establish that observed outcomes were due to the [intervention(s).](#_bookmark8) - A range of approaches exists for improving healthcare. SQUIRE may be adapted for reporting any of these. - Authors should consider every SQUIRE item, but it may be inappropriate or unnecessary to include every SQUIRE element in a particular manuscript. - The SQUIRE Glossary contains definitions of many of the key words in SQUIRE. - The Explanation and Elaboration document provides specific examples of well-written SQUIRE items, and an in-depth explanation of each item. - Please cite SQUIRE when it is used to write a manuscript. |  |
| **Title and Abstract** |  |  |
| **1. Title** | Indicate that the manuscript concerns an [initiative](#_bookmark6) to improve healthcare (broadly defined to include the quality, safety, effectiveness, patient-centeredness, timeliness, cost, efficiency, and equity of healthcare) | Title page, Page 1 |
| **2. Abstract** | 1. Provide adequate information to aid in searching and indexing 2. Summarize all key information from various sections of the text using the abstract format of the intended publication or a structured summary such as: background, local [problem,](#_bookmark10) methods, interventions,   results, conclusions | Abstract, Page 1 |
| **Introduction** | *Why did you start?* |  |
| [**3. Problem**](#_bookmark10) [**Description**](#_bookmark10) | Nature and significance of the local [problem](#_bookmark10) | Introduction – Problem Description, Page 4 |
| **4. Available knowledge** | Summary of what is currently known about the [problem,](#_bookmark10) including relevant previous studies | Introduction –Available Knowledge, Page 4 |

| **5.** [**Rationale**](#_bookmark12) | Informal or formal frameworks, models, concepts, and/or [theories](#_bookmark14) used to explain the [problem,](#_bookmark10) any reasons or [assumptions](#_bookmark0) that were used to develop the [intervention(s),](#_bookmark8) and reasons why the [intervention(s)](#_bookmark8) was  expected to work | Introduction – Rationale, Page 5 |
| --- | --- | --- |
| **6. Specific aims** | Purpose of the project and of this report | Introduction – Specific Objectives, Page 6 |
| **Methods** | *What did you do?* |  |
| **7.** [**Context**](#_bookmark1) | Contextual elements considered important at the outset of introducing the [intervention(s)](#_bookmark8) | Page 7 |
| **8.** [**Intervention(s)**](#_bookmark8) | 1. Description of the [intervention(s)](#_bookmark8) in sufficient detail that others could reproduce it 2. Specifics of the team involved in the work | Page 9 |
| **9. Study of the Intervention(s)** | 1. Approach chosen for assessing the impact of the [intervention(s)](#_bookmark8) 2. Approach used to establish whether the observed outcomes were due to the [intervention(s)](#_bookmark8) | Page 9 |
| **10. Measures** | 1. Measures chosen for studying [processes](#_bookmark11) and outcomes of the [intervention(s),](#_bookmark8) including rationale for choosing them, their operational definitions, and their validity and reliability 2. Description of the approach to the ongoing assessment of contextual elements that contributed to the success, failure, efficiency, and cost 3. Methods employed for assessing completeness and accuracy of data | Page 9 |
|  | 1. Qualitative and quantitative methods used to draw [inferences](#_bookmark5) from the data 2. Methods for understanding variation within the data, including the   effects of time as a variable | Page 9 |
| **12. Ethical**  **Considerations** | [Ethical aspects](#_bookmark2) of implementing and studying the [intervention(s)](#_bookmark8) and how they were addressed, including, but not limited to, formal ethics review and potential conflict(s) of interest | Page 12 |
| **Results** | *What did you find?* |  |
| **13. Results** | 1. Initial steps of the [intervention(s)](#_bookmark8) and their evolution over time (*e.g.*, time-line diagram, flow chart, or table), including modifications made to the intervention during the project 2. Details of the [process](#_bookmark11) measures and outcome 3. Contextual elements that interacted with the [intervention(s)](#_bookmark8) 4. Observed associations between outcomes, interventions, and relevant contextual elements 5. Unintended consequences such as unexpected benefits, problems, failures, or costs associated with the [intervention(s).](#_bookmark8) 6. Details about missing data | Results – Participant quotations embedded throughout Results section, Page 12-18 |
| **Discussion** | *What does it mean?* | Discussion, Page 18 |
| **14. Summary** | 1. Key findings, including relevance to the [rationale](#_bookmark12) and specific aims 2. Particular strengths of the project | Discussion, Page 18 |

| **15. Interpretation** | 1. Nature of the association between the [intervention(s)](#_bookmark8) and the outcomes 2. Comparison of results with findings from other publications 3. Impact of the project on people and [systems](#_bookmark13) 4. Reasons for any differences between observed and anticipated outcomes, including the influence of [context](#_bookmark1) 5. Costs and strategic trade-offs, including [opportunity costs](#_bookmark9) | Discussion, Page 18 |
| --- | --- | --- |
| **16. Limitations** | 1. Limits to the [generalizability](#_bookmark3) of the work 2. Factors that might have limited [internal validity](#_bookmark7) such as confounding, bias, or imprecision in the design, methods, measurement, or analysis 3. Efforts made to minimize and adjust for limitations | Discussion (limitations discussed within reflexive and transferability considerations on Page 18  And also summarized under Strengths and Limitations in Page 2) |
| **17. Conclusions** | 1. Usefulness of the work 2. Sustainability 3. Potential for spread to other [contexts](#_bookmark1) 4. Implications for practice and for further study in the field 5. Suggested next steps | Page 22 |
| **Other information** |  | Declarations-Page 30 |
| **18. Funding** | Sources of funding that supported this work. Role, if any, of the funding organization in the design, implementation, interpretation, and reporting | Declarations – Funding, Page 30-31 |

# Table 2. Glossary of key terms used in SQUIRE 2.0. This Glossary provides the intended meaning of selected words and phrases as they are used in the SQUIRE 2.0 Guidelines. They may, and often do, have different meanings in other disciplines, situations, and settings .

**Assumptions**

Reasons for choosing the activities and tools used to bring about changes in healthcare services at the [system](#_bookmark13) level.

# Context

Physical and sociocultural makeup of the local environment (for example, external environmental factors, organizational dynamics, collaboration, resources, leadership, and the like), and the interpretation of these factors (“sense-making”) by the healthcare delivery professionals, patients, and caregivers that can affect the effectiveness and [generalizability](#_bookmark3) of [intervention(s).](#_bookmark8)

# Ethical aspects

The value of [system-](#_bookmark13)level [initiatives](#_bookmark6) relative to their potential for harm, burden, and cost to the stakeholders. Potential harms particularly associated with efforts to improve the quality, safety, and value of healthcare services include [opportunity costs,](#_bookmark9) invasion of privacy, and staff distress resulting from disclosure of poor performance.

# Generalizability

The likelihood that the [intervention(s)](#_bookmark8) in a particular report would produce similar results in other settings, situations, or environments (also referred to as external validity).

# Healthcare improvement

Any systematic effort intended to raise the quality, safety, and value of healthcare services, usually done at the [system](#_bookmark13) level. We encourage the use of this phrase rather than “quality improvement,” which often refers to more narrowly defined approaches.

# Inferences

The meaning of findings or data, as interpreted by the stakeholders in healthcare services – improvers, healthcare delivery professionals, and/or patients and families

# Initiative

A broad term that can refer to organization-wide programs, narrowly focused projects, or the details of specific interventions (for example, planning, execution, and assessment)

# Internal validity

Demonstrable, credible evidence for efficacy (meaningful impact or change) resulting from introduction of a specific intervention into a particular healthcare [system.](#_bookmark13)

# Intervention(s)

The specific activities and tools introduced into a healthcare [system](#_bookmark13) with the aim of changing its performance for the better. Complete description of an intervention includes its inputs, internal activities, and outputs (in the form of a logic model, for example), and the mechanism(s) by which these components are expected to produce changes in a [system’s](#_bookmark13) performance.

# Opportunity costs

Loss of the ability to perform other tasks or meet other responsibilities resulting from the diversion of resources needed to introduce, test, or sustain a particular [improvement](#_bookmark4) initiative

# Problem

Meaningful disruption, failure, inadequacy, distress, confusion or other dysfunction in a healthcare service delivery [system](#_bookmark13) that adversely affects patients, staff, or the [system](#_bookmark13) as a whole, or that prevents care from reaching its full potential

# Process

The routines and other activities through which healthcare services are delivered

# Rationale

Explanation of why particular [intervention(s)](#_bookmark8) were chosen and why it was expected to work, be sustainable, and be replicable elsewhere.

# Systems

The interrelated structures, people, [processes,](#_bookmark11) and activities that together create healthcare services for and with individual patients and populations. For example, systems exist from the personal self-care system of a patient, to the individual provider-patient dyad system, to the microsystem, to the macrosystem, and all the way to the market/social/insurance system. These levels are nested within each other.

# Theory or theories

Any “reason-giving” account that asserts causal relationships between variables (causal theory) or that makes sense of an otherwise obscure [process](#_bookmark11) or situation (explanatory theory). Theories come in many forms, and serve different purposes in the phases of [improvement](#_bookmark4) work. It is important to be explicit and well-founded about any informal and formal theory (or theories) that are used.
